# Recent progress of in-fiber WGM microsphere resonator

**DOI:** 10.1007/s12200-023-00066-3

**Published:** 2023-05-25

**Authors:** Yong Yang, Zijie Wang, Xiaobei Zhang, Qi Zhang, Tingyun Wang

**Affiliations:** Key Laboratory of Specialty Fiber Optics and Optical Access Networks, Joint International Research Laboratory of Specialty Fiber Optics and Advanced Communication, Shanghai Institute for Advanced Communication and Data Science, Shanghai University, Shanghai, 200444 China

**Keywords:** Whispering gallery mode, Microsphere resonator, In-fiber device, Sensor

## Abstract

In-fiber whispering gallery mode (WGM) microsphere resonators have received remarkable attention due to the superiorities of compact structure, high stability and self-alignment. As an in-fiber structure, WGM microsphere resonators have been demonstrated in various applications, such as sensors, filters and lasers, which have significant impacts on modern optics. Herein, we review recent progress of in-fiber WGM microsphere resonators, which involve fibers of diverse structures and microspheres of different materials. First, a brief introduction is given to in-fiber WGM microsphere resonators, from structures to applications. Then, we focus on recent progresses in this field, including in-fiber couplers based on conventional fibers, capillaries and micro-structure hollow fibers, and passive/active microspheres. Finally, future developments of the in-fiber WGM microsphere resonators are envisioned.

**Graphical Abstract:**

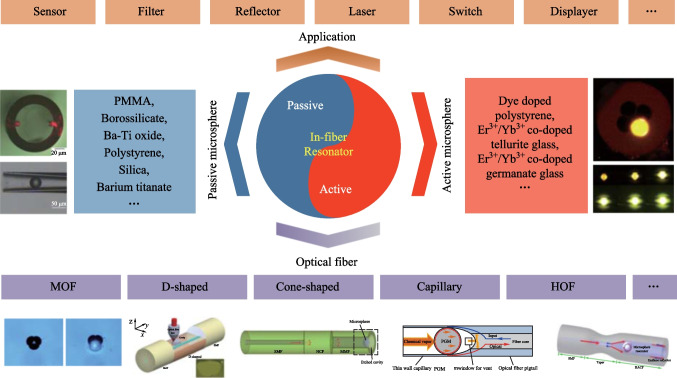

## Introduction

Whispering gallery mode (WGM) microsphere resonators have attracted enormous attention due to their ultrahigh quality (*Q*) factor and small mode volume [1]. By virtue of strong light-matter interaction in such a structure, WGM microsphere resonators show great potential in extensive applications including lasers [2–4], sensors [5–7], nonlinear optics [8], optomechanics [9, 10], orbital angular momentum related applications [11], etc. Before realizing these applications, a significant problem encountered was how to couple light into and out of the WGM microsphere. Heretofore, many coupling structures have been proposed based on evanescent wave coupling, including prism coupling [12–14], D-shaped fiber coupling [15–17], angle-polished fiber coupling [18–20], and tapered fiber coupling [21–23]. Although high coupling efficiency or high flexibility could be achieved based on the above-mentioned methods, long-term stability and precise alignment remain challenging. As an emerging coupling technology, in-fiber coupling has attracted enormous interest [24–27] in recent years, by virtue of self-alignment and high stability. Generally, an in-fiber WGM microsphere resonator is constructed by inserting a microsphere into an optical fiber which acts as an in-fiber coupler for the excitation of WGMs. Earlier reported WGM microsphere resonators utilized the tip of a micro-structured optical fiber (MOF) attaching the microsphere for light coupling [28, 29], and their *Q* factors are relatively low. To enhance the *Q* factor of the resonator and robustness of the coupler, more recent solutions was embedding the microsphere into capillaries [26], MOFs [30] and so on. Meanwhile, chemical etching and femtosecond laser micromachining technology (FLMT) have been employed to modify optical fibers for high-efficiency coupling. Modified conventional fibers, photonic crystal fibers (PCFs) and capillaries [26, 27, 31] as well as D-shaped and exposed-core fibers [32–36] have been proposed, allowing for strong evanescent field interaction. Chemical etching and FLMT are used mainly to form a cone-shaped region in an optical fiber, where the microsphere is inserted and locked. Moreover, some structures based on micro-structured hollow fibers have been proposed and demonstrated to realize in-fiber couplers without the necessity of chemical etching or FLMT, such as suspended core fibers and annular core fibers [37, 38]. In these fibers, the cone-shaped region is usually formed by incomplete collapse of the hollow structure under arc discharge of a fusion splicer or focused light from a CO_2_ laser.

**Fig. 1:**
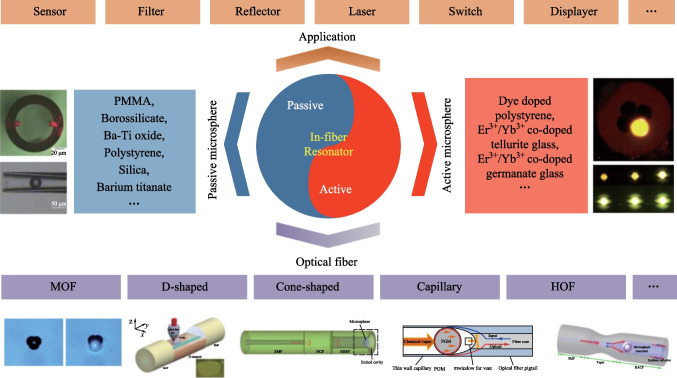
Overall description of in-fiber resonator in terms of fiber structure and resonator material. MOF: micro-structured optical fiber; HOF: hollow optical fiber; PMMA: polymethyl methacrylate. Reproduced with permission from Refs. [16, 24, 25, 27, 42, 46, 49–51]

For microspheres, either passive or active ones with diameter slightly smaller than that of the fiber can be adopted. Passive microspheres made of polymethyl methacrylate (PMMA), polymer, borosilicate, Ba-Ti oxide, polystyrene, silica, barium titanate, and so on, can be embedded into the mentioned optical fibers to construct in-fiber resonators, and have been successfully applied in applications including temperature [37], refractive index [39], and displacement sensing [40]. Besides, dye and rare-earth ion can be doped into the microsphere material, thereby emitting fluorescence [41–45]. As the counterpart of passive microspheres, active microspheres are of great interest for in-fiber resonators because they provide the possibilities of remote excitation and breaking the limitations of passive microspheres. For example, color-tunable luminescence and self-referenced biosensing have been demonstrated by using active microspheres [46, 47]. Additionally, in-fiber laser is a current hot spot and many efforts are being made to optimize the structure of optical fiber and to improve the coupling efficiency of in-fiber coupler [48]. Figure 1 gives an overall description of the in-fiber resonator in terms of optical fibers and microspheres, and the potential applications are also presented.

Due to the flexibility and diversified structures of in-fiber resonators, a variety of optical mechanisms can be exploited, such as Michelson interference, Fano resonance and electromagnetically induced transparency (EIT) [49, 52, 53]. In essence, these optical phenomena derive from the interference between the light reflected in the optical fiber and different WGMs. They expand the application fields of in-fiber resonators and enhance the device performances. For example, additional Michelson interference can be utilized to simultaneously sense other physical parameters except for WGM resonance-based sensing. Reflection can transform the WGM resonance into Fano resonance that can be used in optical switches [54]. Besides, EIT makes the device transparent at special wavelengths and then able to work as band-pass filters [55].

This review gives an overview of the recent progress of in-fiber WGM microsphere resonators. It begins with an introduction to the in-fiber resonator composed of in-fiber coupler and microsphere. Typical optical fibers used in this area include conventional fibers, capillaries and hollow optical fibers with micro-structures, while microspheres can be divided into passive and active microspheres, with dye or rare-earth ion usually used as dopants. With these in mind, then we give some typical examples of various in-fiber couplers based on the aforementioned fibers and typical microspheres embedded in the in-fiber couplers. Moreover, the intrinsic logic and transitional structures between two kinds of in-fiber coupling structures are explained and introduced. Finally, we comprehensively analyze the advantages, disadvantages and current status of in-fiber WGM microsphere resonators, and then discuss the development prospect of the emerging WGM microsphere coupling structures.

## In-fiber coupler based on conventional optical fiber

### D-shaped fiber based couplers

**Fig. 2:**
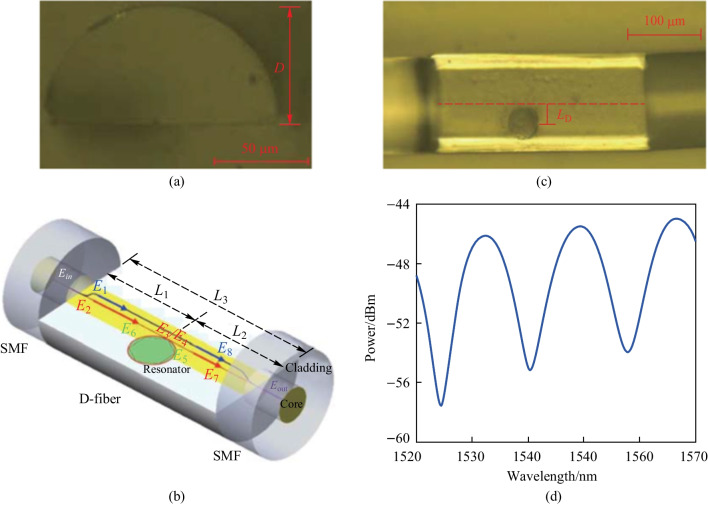
**a** Microscopic image of cross-section of the D-shaped fiber. **b** Schematic of the WGM resonator with D-shaped fiber coupling structure. **c** Microscopic image and **d** transmission spectrum of the D-shaped fiber based coupler with a hole for accommodating microspheres. Reproduced with permission from Ref. [15]

Conventional optical fibers, on its own, have no space to accommodate microspheres, and require mechanical processing to form a suitable spatial structure for embedding microspheres. A common scheme is preparing a D-shaped fiber and then inscribing a certain structure in the D-shaped fiber by FLMT. In 2017, Shi et al. fabricated a D-shaped fiber by side polishing a single-mode fiber (SMF) [15], as shown in Fig. 2a. The diagram of the D-shaped fiber based coupling structure with a hole for embedding the microsphere is shown in Fig. 2b, which was realized by femtosecond laser ablation. The microscopic image of the fabricated structure is shown in Fig. 2c [15]. Without the hole, the structure is a Mach–Zehnder interferometer (MZI) because the input light from SMF is split into two parts and propagates in the air (*E*_1_) and fiber core (*E*_2_), respectively. Figure 2d shows the transmission spectrum of the D-shaped fiber based coupler they obtained, which is a typical spectrum of MZI with two-beam interference characteristics.

**Fig. 3:**
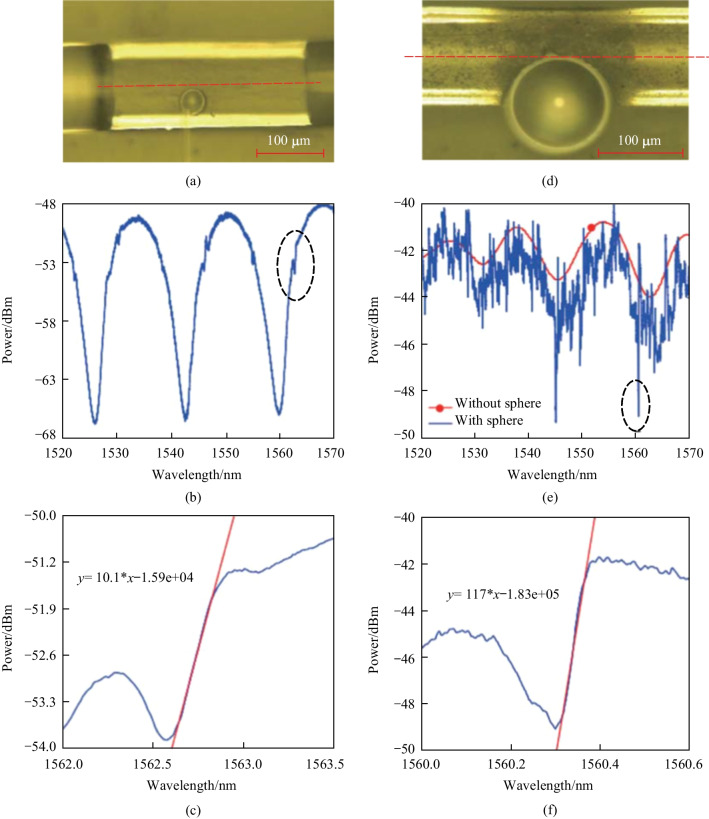
**a** Microscopic image and **b** transmission spectrum of the D-shaped fiber based coupler where the microsphere is ~ 1 μm away from the fiber core. **c** Detailed view of the asymmetric spectrum around 1562.7 nm as shown by the dashed circle in **b**. **d** Microscopic image and **e** transmission spectrum of the D-shaped fiber-based coupler where the microsphere overlaps with the fiber core by ~ 2 μm. **f** Detailed view of the asymmetric spectrum around 1560.3 nm as shown by the dashed circle in **e**. Reproduced with permission from Ref. [15]

The influence of the position of hole was also investigated. Figure 3a shows the microscopic images of the D-shaped fiber based coupler with a PMMA microsphere embedded in the hole, where the diameter of the microsphere is ~ 34.7 μm, and the distance between the microsphere and fiber core is ~ 1 μm. They observed asymmetric line shapes in the transmission spectrum of the in-fiber resonator, as shown in the dashed circle in Fig. 3b and the detailed spectrum around 1562.7 nm shown in Fig. 3c. These asymmetric line shapes were thought to be caused by the coupling between the WGM microsphere resonator and the MZI, and the slope of the asymmetric line shapes was found to be 10.1 dB/nm. They also made a D-shaped coupler with a microsphere of ~ 114 μm in diameter and overlaping the fiber core by ~ 2 μm, as shown in Fig. 3d, the slope of asymmetric line shape increased to 117 dB/nm, as shown in Fig. 3e and f. Hence, it can be concluded that the spatial distance between the fiber core and the microsphere has an effect on the slope of the asymmetric line shape in transmission spectrum, which in turn results in different *Q* factors of the in-fiber resonator.

**Fig. 4:**
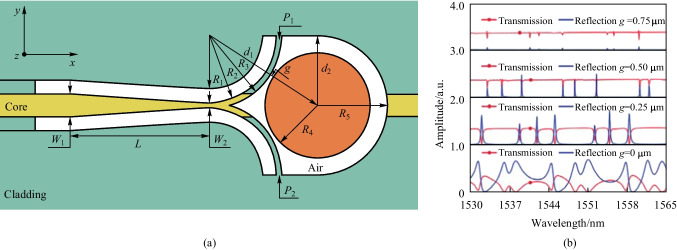
**a** Schematic of the Y-junction coupled WGM resonator. **b** Simulated reflection and transmission spectra when the width of the coupling branch is 1 μm. The transmission spectra are obtained at positions *P*_1_ and *P*_2_, while the reflection spectrum is obtained at the input port. Reproduced with permission from Ref. [57]

Based on the aforementioned D-shaped fiber based coupler, Shi et al. also proposed to enhance the coupling efficiency by forming a tapered fiber core by femtosecond laser ablation [56]. In addition to this kind of straight-through D-shaped fiber based coupling structure, Shi et al. proposed a reflective D-shaped fiber based coupling structure [57], as shown in Fig. 4a. It is characterized by an in-fiber Y-junction consisting of two branches coupled with a microsphere, and the width of coupling branches has an important effect on the resonant characteristics of the microsphere resonator. Besides, the gap between the microsphere and coupling branches also has an effect on the resonant characteristics. It was found by simulation that the maximum resonant strength was achieved when the width is 1 μm and the gap is 0.25 μm.

**Fig. 5:**
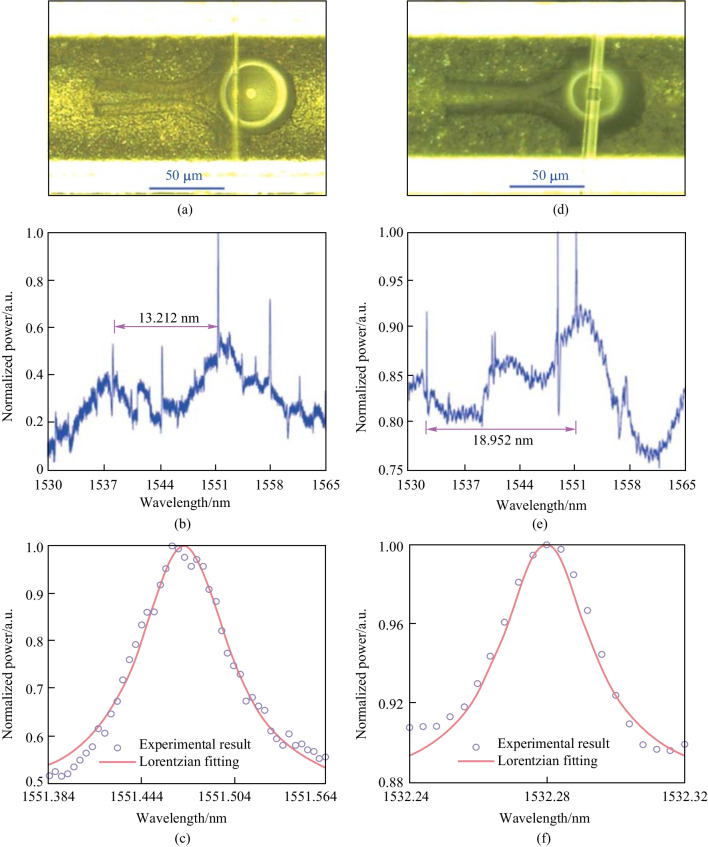
**a** Microscopic image, **b** reflection spectrum, and **c** Lorentzian fitting of the WGM resonance of the in-fiber resonator where a PMMA microsphere with diameter of 39 μm is coupled with the two branches of the Y-junction. **d** Microscopic image, **e** reflection spectrum, and **f** Lorentzian fitting the WGM resonance of the in-fiber resonator where a PMMA microsphere with diameter of 27 μm is coupled with the two branches of the Y-junction. Reproduced with permission from Ref. [57]

The reflective D-shaped fiber based Y-junction coupler was fabricated by laser ablation. The PMMA microsphere was picked up and transferred into a hole between the branches via a tapered fiber. Microspheres with different diameters are investigated, as shown in Fig. 5a and d, where the diameters are 39 and 27 μm, respectively. Figure 5b and e are their reflection spectra and Fig. 5c and f are the corresponding Lorentzian fitting of the WGM resonance around 1551.47 and 1532.28 nm. The in-fiber WGM resonator based on a Y-junction can be utilized as a narrow-band wavelength-selective reflector, and the reflection band can be further narrowed by using a microcavity with higher *Q* factor.

### Cone-shaped fiber based couplers

**Fig. 6:**
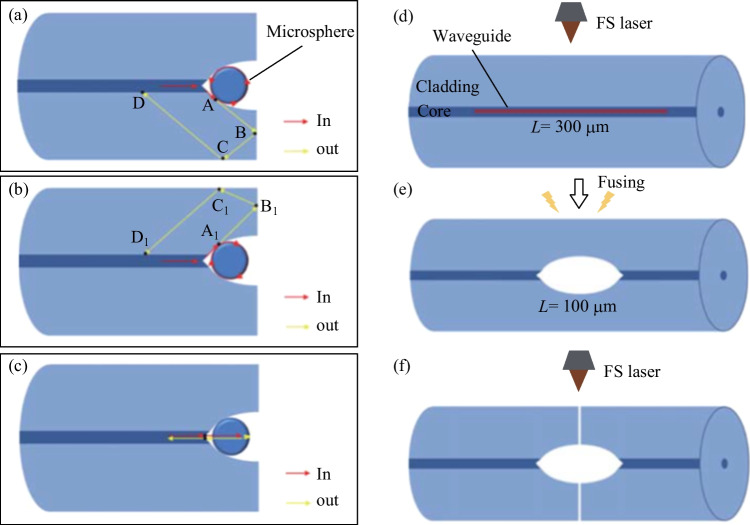
**a**–**c** Three possible optical paths in the cone-shaped fiber based in-fiber resonator. **d**–**f** Schematic of the fabrication process: **d** waveguide inscription, **e** electric arc discharge and **f** carving a scratch. Reproduced with permission from Ref. [59]

In a D-shaped fiber based coupler, a special structure should be inscribed in the D-shaped fiber by FLMT to place the microsphere, and this is a complex micromachining process. A simplified scheme is to directly ablate a hole at the end of a conventional fiber without side-polishing into to D shape [58]. However, for these type of in-fiber resonators, the embedded microsphere is not locked and thus is unstable. To overcome this disadvantage, cone-shaped fiber based in-fiber resonator was proposed by Li et al. in 2022 [59]. As shown in Fig. 6a–c, the structure exhibited three possible optical paths. For the first two optical paths, the light is first coupled into the microsphere and then coupled back to the fiber core, indicating a reflection-type coupling structure. The third optical path forms Fabry–Pérot (FP) interference by reflections at the microsphere front and back surfaces.

**Fig. 7:**
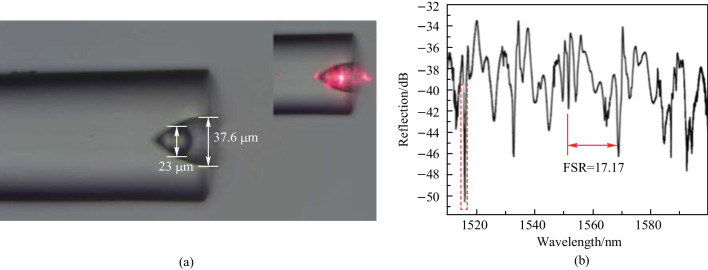
**a** Microscopic image of the cone-shaped fiber based in-fiber resonator. Inset: the device under red light illumination. **b** Reflection spectrum of the device. Reproduced with permission from Ref. [59]

The fabrication process of the cone-shaped fiber based coupler included waveguide inscription, arc discharge and carving a scratch by laser ablation, as shown in Fig. 6d–f. A barium titanate glass microsphere was embedded into the cone and is further fixed by UV glue. The microscopic image of the prepared device is shown in Fig. 7a and the inset shows the device under red light illumination. The reflection spectrum of the device was experimentally obtained, as shown in Fig. 7b. The FSR of the WGM resonant peaks in the spectrum is 17.17 nm.

**Fig. 8:**
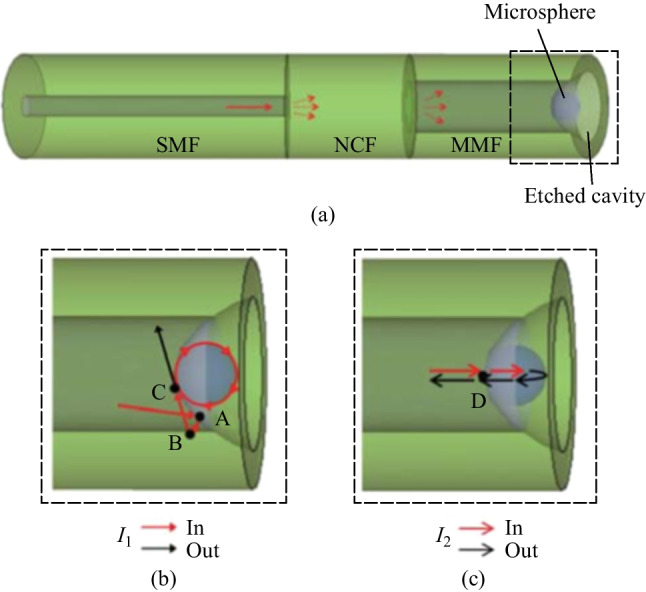
**a** Schematic of the MMF based in-fiber resonator. Optical paths for the **b** WGM and **c** FP mode. Reproduced with permission from Ref. [51]

The above-mentioned in-fiber coupling structure requires micromachining via laser ablation, which is fairly expensive and inconvenient. A similar coupling structure without laser ablation was reported by Bai and Wang in 2018, which is based on an etched cavity at the end of a multimode fiber (MMF) [51]. As shown in Fig. 8a, the whole structure is composed of SMF, no-core fiber (NCF), etched MMF and microsphere. The incident light enters the SMF and propagates along the SMF core and diverges at the interface between SMF and NCF. The light travels along the MMF with multiple modes. There are two possible optical paths for the light arriving at the interface of the etched cavity and the microsphere. In one path, after grazing incidence at the interface of the microsphere and the etched cavity, the light is coupled into the microsphere, exciting the WGMs, and finally coupled out of the microsphere and returns into the fiber core, as shown in Fig. 8b. In contrast, the other optical path forms FP interference by reflections at the microsphere front and back surfaces, as shown in Fig. 8c.

**Fig. 9:**
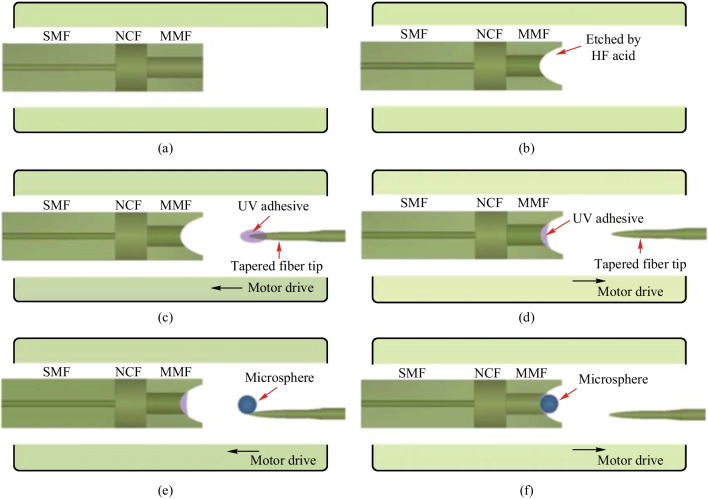
Schematic of the device fabrication process: **a** fusion splice SMF, NCF and MMF; **b** etch the end of MMF; **c**, **d** add UV glue onto the bottom of the cavity; **e**, **f** glue the microsphere to the bottom of the cavity. Reproduced with permission from Ref. [51]

The MMF based in-fiber resonator was fabricated by the process given in Fig. 9. Firstly, one end of a short section of NCF was spliced with SMF and another end with MMF, as shown in Fig. 9a. Secondly, the end of the MMF was etched by the Hydrofluoric acid with concentration of 40% to form a cone-shaped cavity, as shown in Fig. 9b. Thirdly, a thin layer of UV glue was applied to the bottom of the cavity via a tapered fiber tip, as shown in Fig. 9c and d. Finally, the microsphere was glued onto the bottom of the cavity while adjusting its position until WGM resonances appear in the reflection spectrum. The adhesive is then cured by UV light, as shown in Fig. 9e and f.

**Fig. 10:**
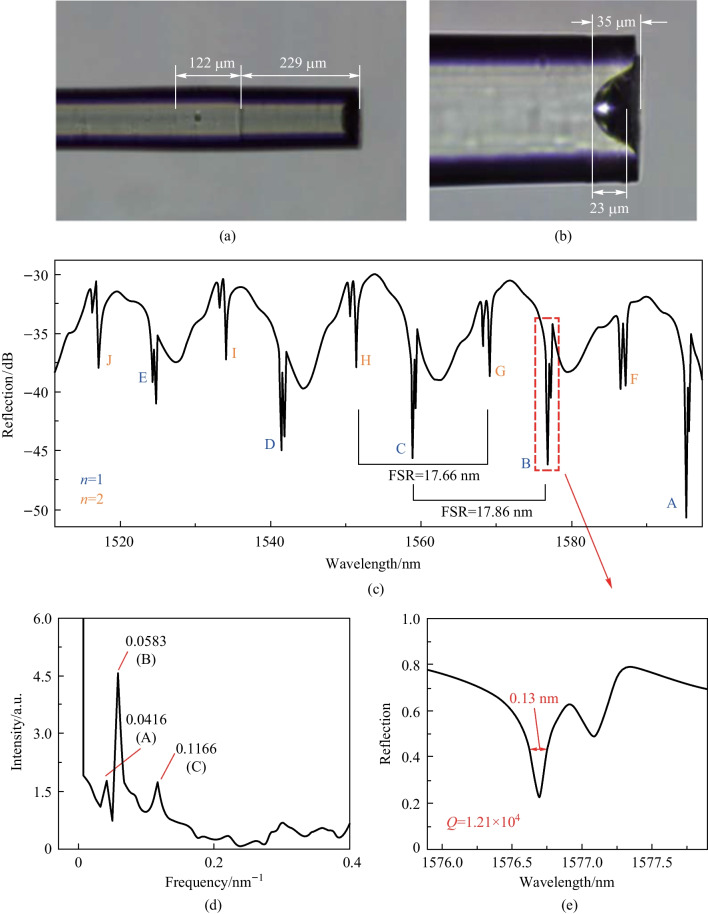
**a** Microscopic image of the device sample of the MMF based in-fiber resonator. **b** Enlarged view of the cavity at the MMF end. **c** Reflection spectrum of the device sample. **d** Spatial frequency spectrum obtained by FFT. **e** The enlarged view of the peak shown in the dashed rectangle region in **c**. Reproduced with permission from Ref. [51]

Microscopic images of a device sample of the MMF based in-fiber resonator they fabricated are shown in Fig. 10a and d where the NCF length is 122 μm, the MMF length is 229 μm, the depth of the cone-shaped cavity is ~ 35 μm and the diameter of the microsphere is ~ 23 μm. The reflection spectrum are displayed in Fig. 10c. They also obtained the corresponding spatial frequency spectrum by fast Fourier transform (FFT) as given in Fig. 10d. It can be found that there are three peaks in the spatial frequency spectrum, which are marked with A, B and C respectively. They investigated the origin of these three peaks and found that the peaks B and C were attributed to the WGM, where the dominant peak B corresponded to the optical path where light circulated once on the inner wall of microsphere while the optical path corresponding to peak C is twice the length of the former one. Through calculation and comparison, they found peak A was generated due to the FP mode. From the enlarged view of the dashed rectangle in Fig. 10c, it can be seen that the full width at half-maximum and *Q* factor are 0.13 nm and 1.21 × 10^4^, respectively, as shown in Fig. 10e. A similar angle polished MMF based in-fiber resonator has been also proposed by Hua and Wang in 2021, which completely wraps around the microsphere via two angle polished and etched MMFs and then protects that from pollution [60].

## Capillary based in-fiber coupler

### Internally etched capillary based couplers

**Fig. 11:**
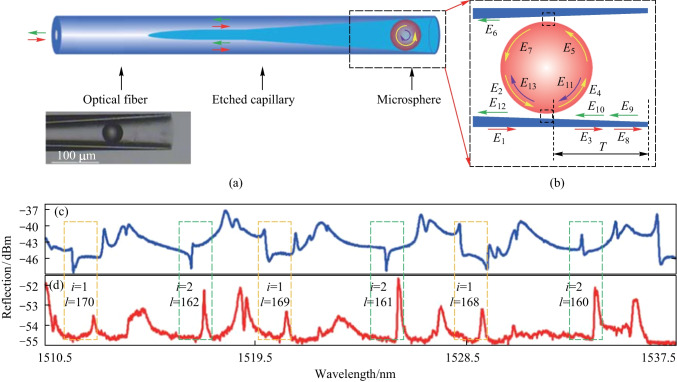
**a** Schematic of the capillary based in-fiber resonator and **b** light propagation in the coupled region. The inset in **a** shows the image of fabricated coupler. The reflection spectra when the front end of device was placed in **c** air, and **d** matching liquid, respectively. Reproduced with permission from Ref. [49]

The above-mentioned MMF-based coupler has an obtuse cone angle, which leads to the FP interference pattern in the reflection spectrum, while capillary with inherent hollow channel is the preferred choice for constructing in-fiber coupler with an acute cone angle [61]. In 2017, Zhang et al. proposed an in-fiber resonator consisting of a small inner diameter capillary spliced with a SMF and a microsphere in the capillary [49], as shown in Fig. 11a. The inner and outer diameters of the capillary are 5 and 125 μm, respectively. They etched a cone-shaped inwall in the capillary to hold the microsphere, and the wall thickness of the frontend of the capillary was ~ 5 μm in the experiment for optimal coupling between the microsphere and the capillary. Figure 11b shows the light propagation in the in-fiber resonator with *E*_*m*_ (*m* = 1–13) the optical field at the specified positions. A barium titanite microsphere with the diameter of 45 μm and refractive index of 1.93 was embedded into the conical structure with assistance of a tapered fiber and two precise 3D translation stages. The microscopic image of this coupler is shown in the inset of Fig. 11a. The cone angle induced by fusion splicing at the interface of SMF and capillary is acute so that reflections can be avoided.

Figure 11c and d show the reflection spectra when the capillary end of the device was placed in air and matching liquid, respectively. Fano resonances was observed in the former case, indicating that the end surface of the capillary acted as a reflector. The Fano line shape transformed into Lorentz line shape of WGM as the device was in the matching liquid, which can be found in the orange and green dashed rectangles marked in Fig. 11c and d, respectively. It can be also found that the resonance wavelengths shown in Fig. 11d have a slight redshift compared with those in Fig. 11c, which was explained by the partial contact between the matching liquid and microsphere.

The traditional WGM exhibits a symmetric Lorentzian line shape, which can transform into an asymmetric Fano line shape when the additional light is introduced due to the special device structure, such as the reflected light from the capillary front end in the above device. This asymmetric profile has a steep slope, which has been intensively investigated in the fields of ultrahigh sensitivity sensing [62, 63], slow light [64], and optical switching [65–67]. Fano resonances have been observed in various WGM microcavity structures, e.g., two directly or indirectly coupled WGM resonators [68–70], a WGM resonator in an aqueous environment [71], a WGM resonator coupled with a grating and an ultrathin tapered fiber [72, 73], etc.

**Fig. 12:**
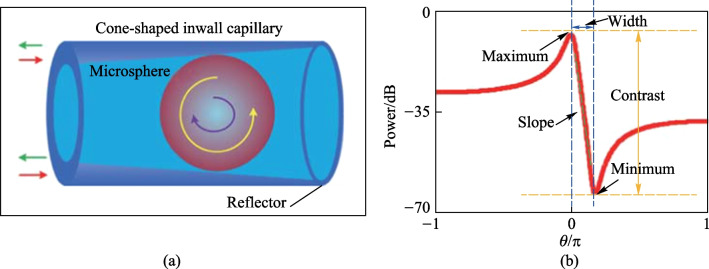
**a** Schematic of the cone-shaped inwall capillary based microsphere resonator. **b** A typical Fano line shape and some defined parameters. Reproduced with permission from Ref. [53]

In-fiber coupler is also an ideal platform for the investigation of Fano resonance. Zhang et al. theoretically investigated the cone-shaped inwall capillary based in-fiber resonator they developed [53]. Figure 12a gives the model of the in-fiber resonator and some parameters of Fano resonance is defined in Fig. 12b [53]. The normalized reflection *P*_R_ can be expressed as

1 $${{P}_{\text{R}}}={{\left| r{{\left(\frac{t-\tau t{{p}^{2}}}{1-\tau {{t}^{2}}{{p}^{2}}}\right)}^{2}}\exp (2\text{i}\delta )-\frac{\sqrt{\tau }{{k}^{2}}p}{1-\tau {{t}^{2}}{{p}^{2}}} \right|}^{2}},$$

where *t* and *k* are the transmission and coupling coefficients of the device, satisfying *t*^2^ + *k*^2^ = 1. *τ* is the loss coefficient, and *r* is the reflectivity at interface of capillary and air. *p* = exp(i*θ*/2) is halfway phase factor and *θ* is the normalized frequency. *δ* = *βL* is the phase difference, where *β* is the propagation constant and *L* is the distance from the capillary-resonator coupling region to the front end of the capillary.

**Fig. 13:**
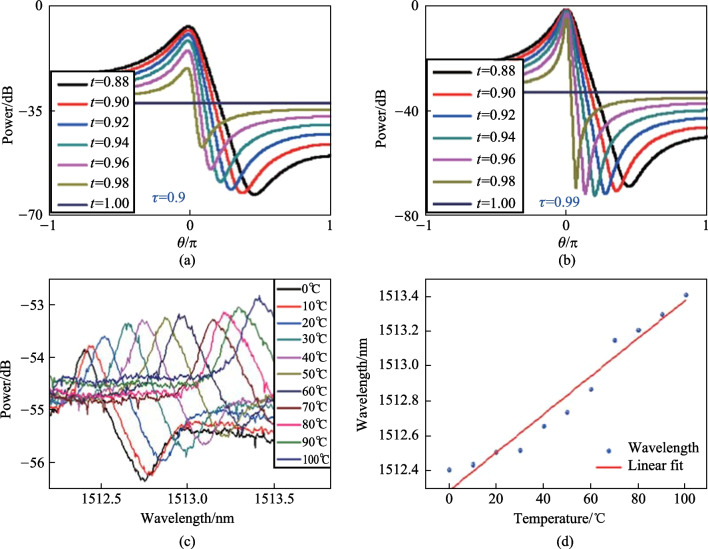
Simulation results when *τ* were **a** 0.9 and **b** 0.99, respectively. **c** Shift of Fano resonance with changing temperatures. **d** Resonant wavelength as a function as temperature. Reproduced with permission from Ref. [53]

Equation (1) shows that the evolution of Fano resonance is a function of *δ, t* and *r*. The simulation in Ref. [53] revealed that *δ* only affected the shape of Fano resonance, but has no effect on the maximum and minimum. Both Lorentzian and Fano line shapes depended on *r*. In addition, the slope of Fano resonance is a vital parameter, which was investigated by varying *t* from 0.88 to 1 for different *τ*. The simulation results were shown in Fig. 13a and b, indicating that the slope of Fano resonance can be optimized by changing the transmission coefficient and loss coefficient.

In the experiment described in Ref. [53], the device was constructed by inserting a barium titanite glass microsphere with a diameter of 45 μm into the capillary with cone-shaped inwall. The Fano resonance was excited and the temperature characteristics were investigated by placing the device into a temperature cabinet. As shown in Fig. 13c and d, the Fano resonance shifts to the longer wavelength when the temperature increases, and the sensitivity is 10.9 pm/°C.

**Fig. 14:**
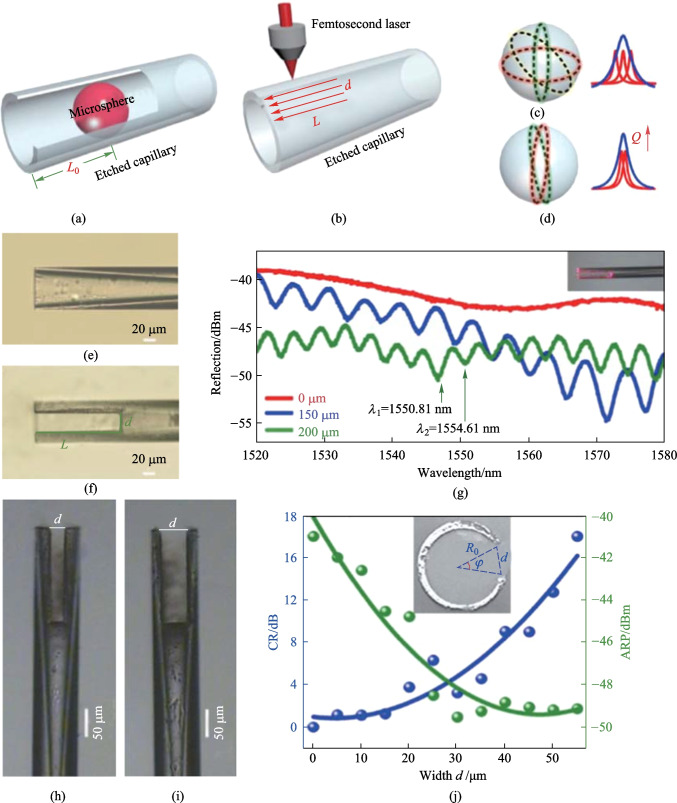
Schematic of the **a** coupling structure and **b** micromachining. Lots of coupling paths exist in **c** and less in **d**. The images of devices **e** before and **f** after micromachining. **g** Reflection spectrum of the coupler with the inscribed length *L* as 0, 150, and 200 μm. The images of the devices with inscribed width *d* as **h** 20 μm and **i** 40 μm and *L* as 150 μm. **j** CR and APR as a function of the inscribed width *d*. Reproduced with permission from Ref. [52]

Although a compact and stable structure can be achieved by the above mentioned scheme, the multiple touching points between the inwall of capillary and the inserted microsphere cause complex spectrum and deteriorated *Q* factor [27]. This problem can be alleviated by adopting FLMT to inscribe a rectangle notch on the etched capillary [52]. Figure 14a and b show the schematics of coupling structure and micromachining process proposed by Zhang et al. in 2018 [52]. *L* and *d* indicate the length and width of the inscribed notch, respectively. Figure 14c and d show the coupling paths in the microsphere before and after femtosecond laser inscription. The reduced number of coupling paths and the narrower full width at half maximum (FWHM) was achieved after micromachining, indicating a higher *Q* factor for the reduced number of coupling paths. The microscopic images of coupler before and after inscription are shown in Fig. 14e and f, respectively. The reflection spectra with *d* of 35 μm, and *L* of 0, 150, and 200 μm, are shown in Fig. 14g. When the coupler is illumined by the red light as shown in the inset of Fig. 14g, Michelson interference pattern was observed, as can be verified by the free spectrum range (FSR). Two parameters, namely the contrast ratio (CR) and the average reflection power (APR), were introduced to characterize the couplers. Figure 14h and i show the images of two couplers with different notch widths *d* of 20 and 40 μm, respectively. The dependences of CR and APR on the notch width were plotted as given in Fig. 14j, indicating that the APR decreases as *d* increases, while the CR increases as *d* increases.

**Fig. 15:**
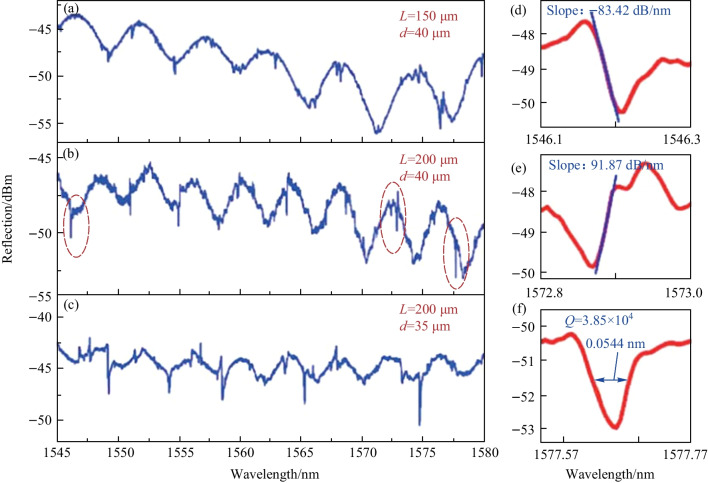
Reflection spectra of the devices with different notch length and width. **a** *L* = 150 μm, *d* = 40 μm. **b** *L* = 200 μm, *d* = 40 μm. **c** *L* = 200 μm, *d* = 35 μm. **d**, **e** The asymmetric Fano resonance with slope of − 83.42 and 91.87 dB/nm, respectively. **f** Symmetric Lorentzian line shape with *Q* factor of 3.85 × 10^4^. Reproduced with permission from Ref. [52]

In their experiment [52], a barium titanite glass microsphere with diameter of 45 μm and refractive index of 1.93 was employed, and the reflection spectra are displayed in Fig. 15. Both the symmetric Lorentzian line shape and asymmetric Fano line shape were observed. As shown in Fig. 15a and b, the ARP decreases when notch length *L* increases, which is in accordance with their theoretical prediction. In addition, they demonstrated that the increased notch width *d* reduced the number of coupling paths, resulting in an increased *Q* factor and slope of Fano resonance, but loss also became higher, as shown in Fig. 15b and c. Figures 15d–f are the enlarged views of the circled resonances in Fig. 15b.

### Half collapsed capillary based couplers

**Fig. 16:**
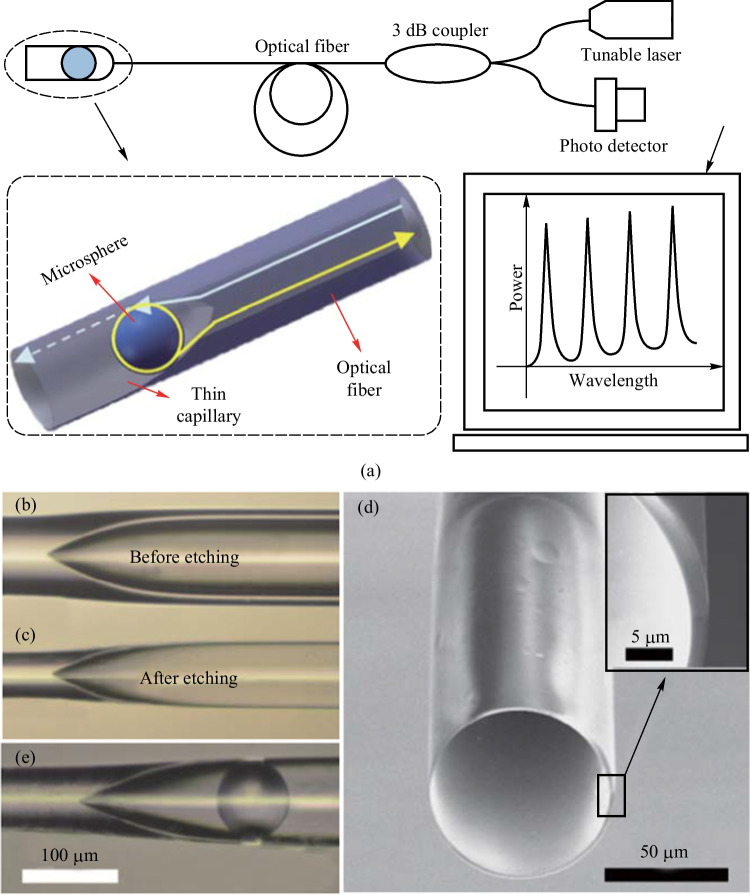
**a** Schematic of the half-collapsed capillary-based in-fiber resonator and experimental setup. Microscopic images of the coupler; **b** before and **c** after etching. **d** SEM image of the etched capillary with a wall thickness of ~ 2 μm. **e** Microscopic image of etched capillary coupled with a microsphere. Reproduced with permission from Ref. [26]

For the above-mentioned small-inner-diameter capillary based in-fiber couplers, the conical structure to accommodate the microsphere was realized by chemical etching. In addition, the conical structure can also be realized by the half collapse of the large-inner-diameter capillary via the arc discharge, which is a relatively simple scheme. In 2013, Wang et al. proposed a half-collapsed capillary based in-fiber resonator [26], and the experimental setup for observing the reflection spectrum are shown in Fig. 16a. The in-fiber coupling structure was composed of MMF and a capillary with an inner diameter of 75 μm and an outer diameter of 125 μm. They were fusion spliced with appropriate welding parameters to produce a conical structure resulted from half-collapse, as shown in Fig. 16b. To enhance the evanescent field at the inner surface of the capillary, the capillary wall was etched from the outside to reduce the wall thickness, as shown in Fig. 16c. The capillary was sealed by fusion splicing it with a short section of optical fiber during etching, and was cleaved after etching, as shown in Fig. 16d. Finally, a microsphere was then fed into the capillary till it was locked at the conical structure, as shown in Fig. 16e.

**Fig. 17:**
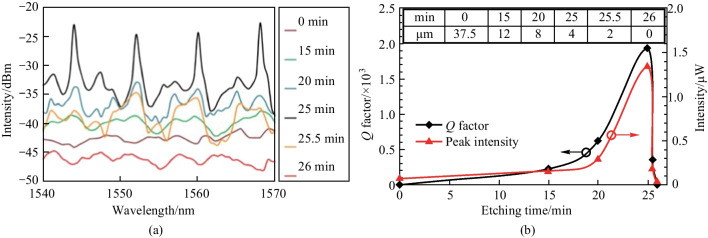
**a** Reflection spectrum evolution with the etching time. **b** Dependence of wall thickness and *Q* factor on the etching time. Reproduced with permission from Ref. [26]

The influence of the wall thickness of the capillary was investigated. The evolution of the reflection spectrum as the etching time increases was displayed as shown in Fig. 17a. When the etching time reaches 15 min, the periodical WGM peaks started to appear. For the etching time of 25 min, the intensity of the WGM peak reached the maximum value where the wall thickness is ~ 4 μm, as shown in Fig. 17b. However, the WGM peaks disappeared when etching time was 26 min, indicating the capillary wall has been etched through. Figure 17b displays that both the *Q* factor and peak intensity have a peak value around the etching time of 25 min.

**Fig. 18:**
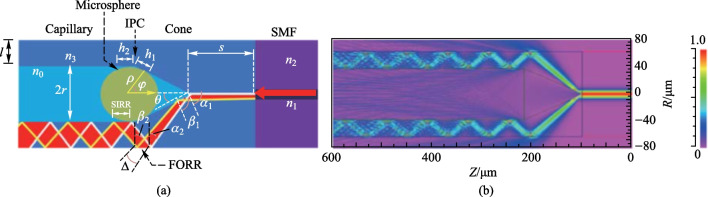
**a** Schematic and **b** simulation result of the capillary-based in-fiber coupler with zigzag transmission. Reproduced with permission from Ref. [77]

The above-mentioned capillary-based in-fiber couplers either need etching or FLMT, which not only introduces additional fabrication processes but also reduces the robustness of coupler. Moreover, all of these coupling structures only involve evanescent wave coupling without consideration of free space coupling [74–76]. Free space coupling is relatively simple because it does not need to generate evanescent waves. In 2020, Yang et al. proposed an in-fiber coupler with optical zigzag transmission to solve these problems [77]. It consisted of SMF, a capillary with inner diameter of 75 μm and outer diameter of 125 μm, and a deformed microsphere. Unlike the aforementioned coupling structures, neither etching nor FLMT was required. The schematic of structure is shown in Fig. 18a, and the simulated optical zigzag transmission by Rsoft is shown in Fig. 8b.The zigzag transmission only exists when the beam width is much smaller than that of its transmission medium, and it is induced by the multimode interference in terms of optical mode. The WGMs of the microsphere can be excited by either evanescent wave coupling or free space coupling, depending on the cone-apex angle *θ* of the device.

**Fig. 19:**
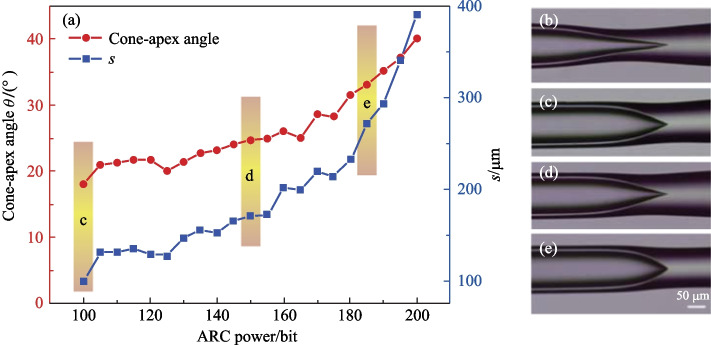
**a** Dependence of cone-apex angle *θ* and complete collapse length *s* on ARC power. **b**–**e** Fabricated device with different *θ* as 9°, 18°, 25°, 33°, respectively. Reproduced with permission from Ref. [77]

**Fig. 20:**
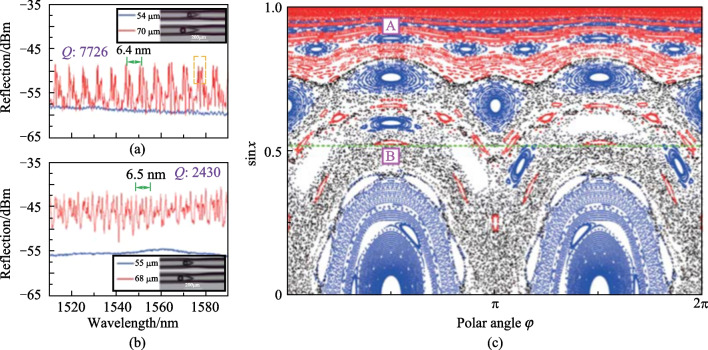
Reflection spectra of the device with the cone-apex angle of **a** 18°, **b** 25°. **c** PSS of a deformed microsphere resonator with deformation parameter as 0.07. Reproduced with permission from Ref. [77]

In Ref. [77], the cone-shaped region in the structure was derived from half-collapse. The cone-apex angle *θ* depended on the collapse velocity related to the arc power. Figure 19a shows the dependences of cone-apex angle *θ* and complete collapse length *s* of the capillary on arc power, revealing that the cone-apex angle *θ* becomes larger as arc power increases. Figure 19b–e show the microscopic images of the devices with *θ* of 9°, 18°, 25° and 33°, respectively. The cone-apex angle *θ* has great influence on the coupling mechanism in this structure, and the reflection spectra for cases of 18° and 25° were obtained as shown in Fig. 20a and b. For the case of *θ* = 18°, the envelop of the reflection spectrum shows a clear FSR with dense peaks near a high-intensity peak, which forms a group of high-intensity WGM peaks, corresponding to the evanescent coupling. For the case of *θ* = 25°, no clear FSR can be found in envelope but sparsely distributed resonance peaks with fairly uniform intensities, which corresponds to free space coupling.

The features of inner ray dynamics in the deformed microsphere resonator was revealed by the Poincare surface of section (PSS). Figure 20c shows the PSS of a microsphere with a deformation parameter of 0.07, where *χ* means the incident angle of the light that reflects and circulates on the inwall of the microsphere, *φ* the polar angle representing the location of reflection point., Three structures, namely chaotic orbits, islands and Kolmogorov-Arnold- Moser (KAM) curves as indicated in black, blue and red was found in the PSS. The chaotic orbits are also named chaotic sea, and islands and KAM curves are both regular orbits where high *Q* factor WGMs are localized.

For the evanescent wave coupling, the initial phase was found to be located around Point A in the PSS where there are dense regular orbits but no chaotic sea. This leads to the spectrum shown in Fig. 20a. On the contrary, for the free space coupling, the initial phase was found to be located around point B in the chaotic sea where sparse regular orbits existed all around. They attributed the sparsely distributed WGM resonance peaks shown in Fig. 20b to the coupling of chaotic orbits with regular orbits or WGMs by dynamical tunneling.

**Fig. 21:**
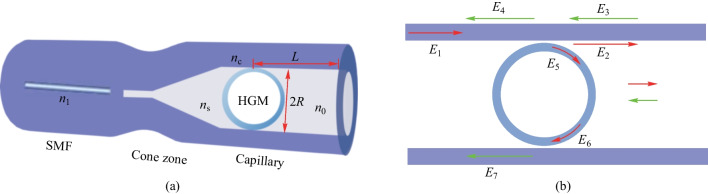
Schematic of **a** half-circle interferometer and **b** optical path in the structure. Reproduced with permission from Ref. [78]

In 2021, Sun et al. proposed a similar structure where the solid microsphere was replaced by a hollow one [78], leading to a different interference mechanism. Based on this kind of in-fiber coupling structure, a half-circle interferometer was introduced and demonstrated. The proposed structure consists of a lead-in SMF spliced with a capillary with inner and outer diameters as 75 and 125 μm, respectively, and a hollow glass microsphere (HGM) with 1–2 μm wall thickness, as shown in Fig. 21a. Figure 21b shows the optical path in the HGM-coupled half-collapsed capillary based in-fiber resonator.

In such a structure, the input light field *E*_1_ launching from SMF enters the capillary via the cone zone. A portion of the light, denoted by *E*_2_, propagates along the capillary wall and is reflected by the end face, whereas the other portion denoted by *E*_5_ couples into the HGM and travels along the HGM wall. At bottom coupling region, the light field *E*_6_ partly couples out of the HGM and is denoted as *E*_7_; the remaining portion continues to travel around the HGM. WGM cannot be excited in the HGM because the propagation constants of capillary’s mode and HGM’s mode are mismatched. Consequently, the reflected light only comprises *E*_7_ and *E*_4_; the reflection spectrum can be treated as a consequence of two-beam interference. This coupler is an ideal platform to realize hydrostatic pressure sensors as deformation of HGM and capillary under pressure can be utilized, which is distinguished from those that utilize the variation of effective refractive index. Sun et al. also demonstrated high-sensitivity hydrostatic pressure sensing based on the proposed in-fiber coupler.

**Fig. 22:**
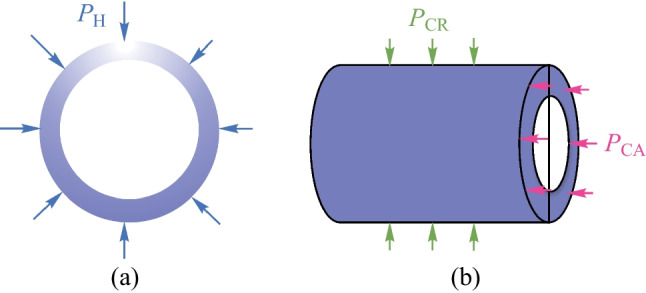
Schematic of pressure on **a** HGM and **b** capillary. Reproduced with permission from Ref. [78]

The process they used to fabricate the coupler was basically the same as for the coupler in Ref. [77]. Moreover, the cone zone was discharged and pulled via a fiber fusion splicer to decrease the cone-apex angle for efficient coupling. As shown in Fig. 22, three kinds of hydrostatic pressure were considered when investigating the sensing characteristics: the pressure on HGM from the surrounding *P*_H_, axial pressure of the capillary *P*_CA_ and radial pressure of the capillary *P*_CR_. The *P*_H_ has influence on HGM, leading to the decreased microsphere radius *R*. *P*_CA_ causes the decreased length *L* and increased inner diameter of capillary, while *P*_CR_ causes increased *L* and reduced inner diameter of capillary. After the theoretical analysis, it was determined that the variation of capillary length *L* caused by *P*_CA_ was the dominating factor for wavelength variation. In addition, the absolute value of sensitivity was inversely proportional to the capillary length *L*.

**Fig. 23:**
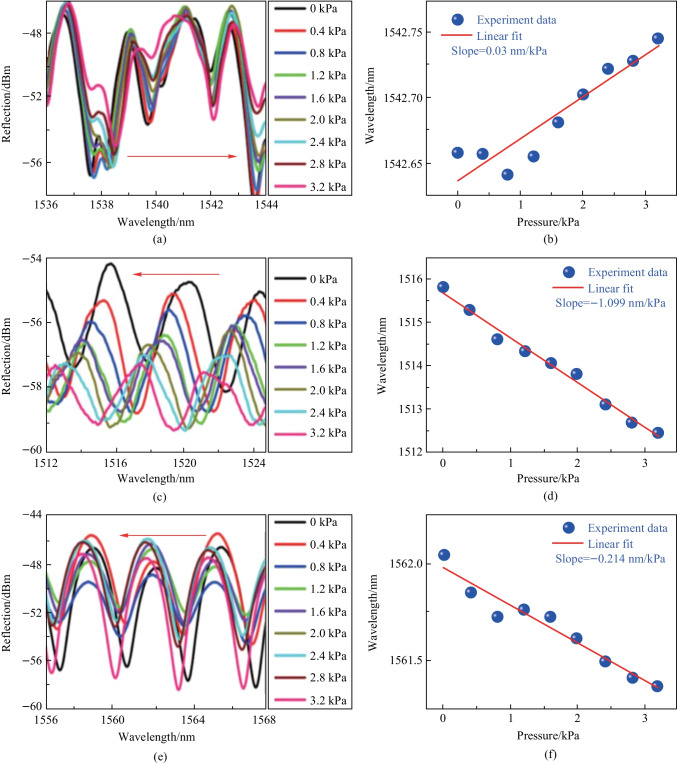
Experimental plots under different hydrostatic pressure with solid barium titanate glass microsphere in **a**, and HGM in **c** and **e**. The initial length *L* of capillary in **c** and **e** are 200 and 270 μm, respectively. **b**, **d**, **f** are the linear fitting of **a**, **c**, **e**, respectively. Reproduced with permission from Ref. [78]

In the experimental described in Ref. [78], a solid barium titanate glass microsphere with a diameter of 71.57 μm was first inserted into capillary cone zone to sense the hydrostatic pressure via WGM mechanism, where the obtained sensitivity was 0.03 nm/kPa as indicated by Fig. 23a and b. To verify the sensing advantage of HGM based on the half-circle interference mechanism, the solid barium titanate glass microsphere was then replaced by a HGM. The hydrostatic pressure sensing performance of the HGM was presented as shown in Fig. 23c–f, the device length *L* was 200 μm in (c) and (d) and 270 μm in (e) and (f). The obtained sensitivities for the two devices were − 1.099 and − 0. 214 nm/kPa, respectively, which highlighted the superiority of the half-circle interference in the HGM for hydrostatic pressure measurement. It was also found that the absolute value of sensitivity was inversely dependent on the initial length *L* of the device.

## Micro-structure hollow fiber based in-fiber coupler

### Hollow annular core fiber based coupler

**Fig. 24:**
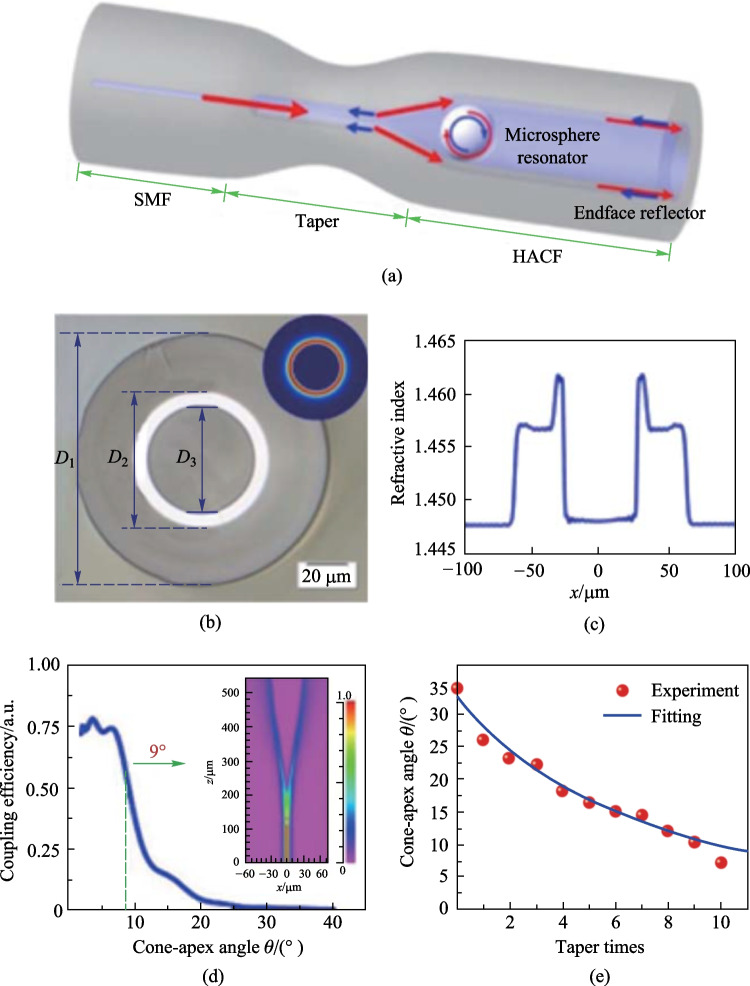
**a** Schematic of the tapered HACF coupled microsphere resonator. **b** Cross-sectional view with the inset as the simulated mode field distribution. **c** Refractive index profile of the HACF. **d** Simulation results of the coupling efficiency as a function of cone-apex angle. The inset in **d** shows the energy distribution in the device at 1550 nm, where most of the energy is confined in the annular core. **e** Experimental results and fitting curve of the cone-apex angle versus taper times. Reproduced with permission from Ref. [50]

Compared with capillaries with simple-structure, hollow fibers with micro-structure can improve the performances of in-fiber couplers in certain aspect and new optical mechanisms can be utilized. The hollow annular core fiber (HACF) has an inner annular high-index core to confine light near the air core, which has been utilized to demonstrate simultaneous measurement of axial strain and temperature in Ref. [38]. Additionally, HACF has the hollow region to contain the microsphere for WGM excitation. In 2018, Wang et al. proposed a tapered HACF based in-fiber WGM resonator, which is composed of SMF, HACF, and a barium titanate glass microsphere, as shown in Fig. 24a [50]. The cross-sectional view and refractive index profile of HACF they proposed are shown in Fig. 24b and c, respectively. The HACF had a low-refractive-index silica cladding with diameter *D*_1_ of 125 μm, a high-refractive-index doped silica annular core with diameter *D*_2_ of 66 μm, and an air core with diameter *D*_3_ of 54 μm. Their refractive indexes were 1.457, 1.462, and 1, respectively. The inset of Fig. 24b shows the simulated cross-section profile of the fundamental mode.

They found that the cone-apex angle of coupler must be controlled because the light directly transmitted into the air core when the cone-apex angle was too large. They intuitively tapered the cone zone for decreasing the angle. The simulation of coupling efficiency as a function of cone-apex angle was conducted, as displayed in Fig. 24d. They also experimentally investigated the dependence of cone-apex angle on taper length, which is proportional to the times of tapering operation. As shown in Fig. 24e, the fitting curve derived from the volume conservation law is well consistent with the experimental result. From these results, they found that the cone-apex angle must be below 9° to obtain a high coupling efficiency, reason was attributed to that fact that the total reflection condition at the interface of cladding and annular core is destroyed when the angle exceeds 9°. Moreover, the small peaks of the coupling efficiency for cone-apex angle less than 9°, as presented in Fig. 24d, was thought to be resulted from the periodic reflection of light in the annular core, which led to a damping oscillation function related to the propagation length.

**Fig. 25:**
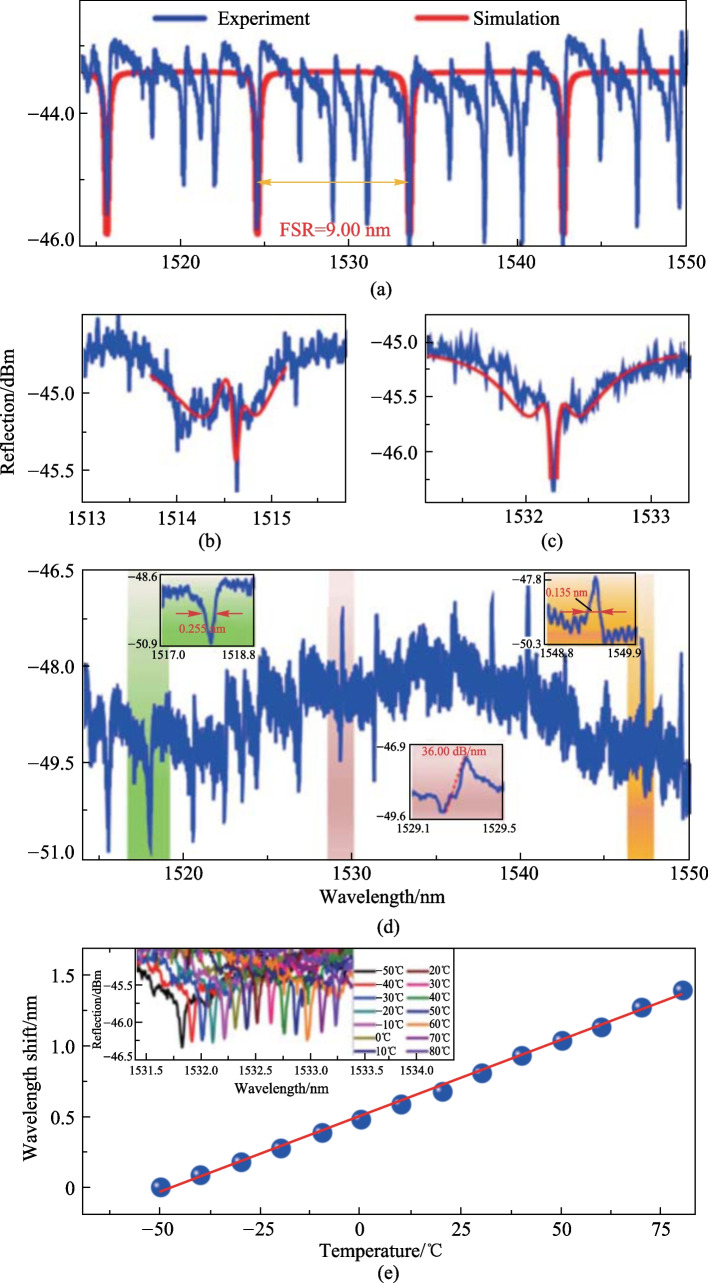
**a** Simulation and experiment spectra when microsphere is close to end face. **b**, **c** Two specific line shapes. **d** Spectrum when microsphere is locked in the HACF. **e** Dependence of wavelength shift on temperature. Reproduced with permission from Ref. [50]

In Ref. [50], a barium titanate glass microsphere with a diameter of 42.3 μm was placed at the position close to the end face of HACF. The simulated and experimental reflection spectra were then obtained as shown in Fig. 25a. The Lorentzian line shape with *Q* factor of 1.3 × 10^4^ was obtained, and the measured FSR was 9 nm which matched well with the diameter of microsphere. It can be found in Fig. 25a that the simulation results agree well with the fundamental WGM modes in the experimental spectrum. The additional resonance peaks between two adjacent fundamental mode peaks was attributed to high-order WGMs. Subsequently, a microsphere with a slightly larger diameter of 42.6 μm was inserted near the end face of HACF and the results are shown in Fig. 25b and c. Both the line shapes were found to be formed by a higher *Q* factor mode located within a lower *Q* factor mode, but with different resonance wavelength detunings. When the microsphere was locked in the cone zone of HACF, both the symmetric Lorentzian and asymmetric Fano line shapes was obtained, as shown in Fig. 25(d). The slope of Fano line shape around 1529.3 nm is about 36.0 dB/nm.

The temperature characteristic of the fabricated device was obtained experimentally by placing the device into a temperature cabinet. In the experiment, the resonance wavelength shifted to the longer wavelength when the temperature increased from − 50 °C to 80 °C with an interval of 10 °C increment, as shown in Fig. 25e. They believed that both the thermal expansion and thermo-optical effect cause wavelength shifts, and the obtained sensitivity was 10.8 pm/°C. They also found that each WGM dip or peak nearly had the same temperature sensitivity. To increase the temperature sensitivity, the PMMA microsphere can be employed [79]. Due to the robust HACF coupling structure, the device has a good stability even after several temperature changes and vibration.

### Suspended dual core hollow fiber-based couplers

**Fig. 26:**
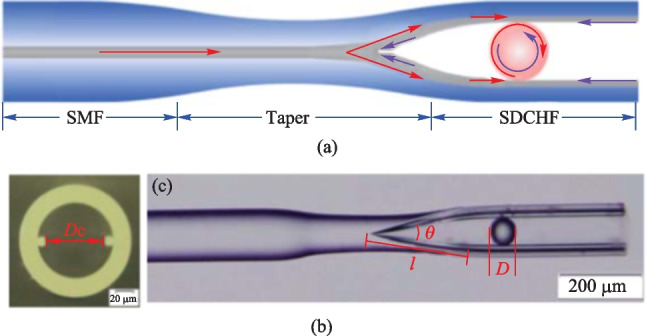
**a** Schematic of the WGM microsphere resonator in a SDCHF. Microscopic images of **b** SDCHF cross section and **c** the SDCHF based device. Reproduced with permission from Ref. [39]

The suspended dual-core hollow fiber (SDCHF) is another promising candidate for constructing in-fiber couplers, due to the hollow structure for embedding the microsphere without requirement of additional processes. Additionally, it has the advantage of reduced coupling paths and has shown potentials in temperature sensing [37]. In 2020, Yang et al. proposed a SDCHF based in-fiber resonator consisting of SMF, SDCHF, and a barium titanate glass microsphere, as shown in Fig. 26a [39]. The SDCHF comprises a cladding with diameter of 125 μm, an air hole with diameter of 83.5 μm, and two suspended cores with distance *D*_c_ of 63.5 μm. The cross section of the SDCHF and the device they fabricated are shown in Fig. 26b and c, respectively.

**Fig. 27:**
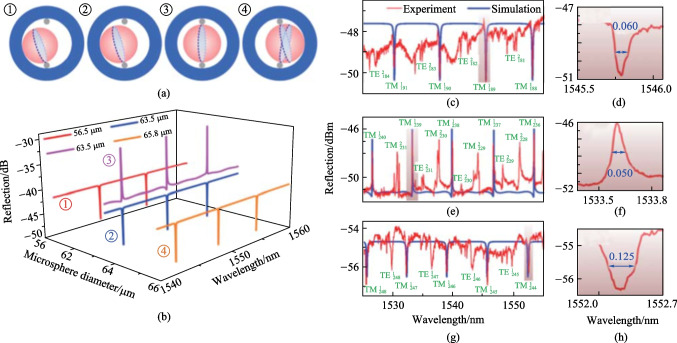
**a** Different coupling situations caused by the diameter discrepancy of the microsphere resonator. **b** Evolution of spectrum as microsphere diameter varies. The obtained experiment and simulation spectra under the microsphere diameter of **c** 51 μm, **e** 63.5 μm and **g** 65.5 μm. **d**, **f**, **h** show the enlarged views of **c**, **e**, **g**. Reproduced with permission from Ref. [39]

In this structure, the optimized coupling efficiency was also achieved by adjusting the cone-apex angle, and the cone-apex angle for the maximum coupling efficiency was found to be in the range of 8° to 15°, which was obtained by simulation and verified experimentally. They also found that size variation of the microsphere would cause different coupling situations, as shown in Fig. 27a. In the cases of ① and ② where the diameters of the microspheres are 56.5 and 63.5 μm, respectively, only one of the cores couples with the inserted microsphere. On the contrary, case ③ exhibits a special situation where the microsphere with diameter of 63.5 μm is wedged between two cores, which can be regarded as the case of an add-drop filter. In the case of ④ where the diameter of microsphere is about 65.8 μm, the microsphere is wedged between the two cores and the inwall of the SDCHF. Multiple coupling paths exist, and the system was regarded as an all-pass structure. As add-drop structure exhibits Fano line shape while the all-pass structure exhibits Lorentzian line shape, the results shown Fig. 27b agrees with the expectation.

They measured the reflection spectra after embedding a microsphere in the cone zone of SDCHF. Figure 27c shows the experiment result for a microsphere with diameter of 51 μm. It was found that the results corresponded to case ①, by matching the experimental curve with the simulation results given in Fig. 27b. A Lorentzian dip with FWHM as 0.06 nm was obtained and the *Q* factor was about 2.5 × 10^4^, as shown in Fig. 27d. When a microsphere with diameter of 63.5 μm was embedded, Lorentzian dip corresponding to case ② was found. It transformed to a Fano line shape after adjusting the coupling position, as displayed in Fig. 27e, corresponding to case ③. Figure 27f shows a Fano resonance extracted from Fig. 27e with a *Q* factor of 3.2 × 10^4^, which resembles a Lorentzian peak because the phase difference in their experiment is close to 0.5π. When the diameter of the microsphere further increased to 65.8 μm, the results they obtained are given in Fig. 27g and h, corresponding to the case ④. The reflection spectrum reverts to Lorentzian dips, but with smaller FSR and doubled FWHM. The former can be attributed to the larger diameter of the microsphere, while the latter is due to the coupling between the microsphere and the two cores of the SDCHF instead of one core.

**Fig. 28:**
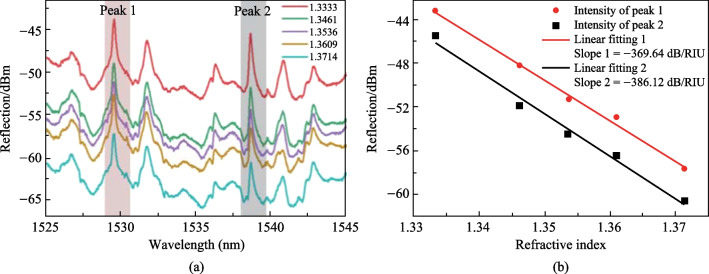
**a** Reflection spectrum versus the RI. **b** Dependence of the reflection intensity on RI. Reproduced with permission from Ref. [39]

The application of this in-fiber resonator as a refractive index (RI) sensor was then demonstrated by contacting the device with glycerol solutions whose RI is in the range of 1.3333 to 1.3714. As shown in Fig. 28a, the intensity of the reflection spectrum decreases as the RI increases, as more light is refracted into the solution from the device. Two WGM peaks as marked in Fig. 28a were further investigated and the dependences of the reflection intensities of on RI are shown in Fig. 28b, where RI sensitivities were found to be − 369.64 and − 386.12 dB/RIU, respectively.

**Fig. 29:**
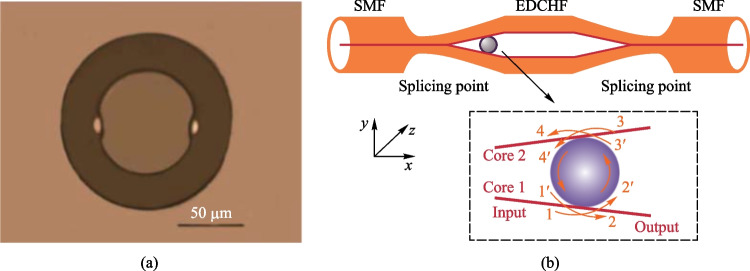
**a** Microscopic image of the cross section of EDCHF. **b** Schematic of the EDCHF based in-fiber resonator. Inset: the optical path. Reproduced with permission from Ref. [37]

The WGM peaks of a SDCHF based in-fiber resonator appear not only in the reflection spectrum but also in the transmission spectrum [80]. In 2018, Zhang et al. prepared a SDCHF with suspended cores almost buried in the cladding, which is also called embedded dual-core hollow fiber (EDCHF) [37], as shown in Fig. 29a. There is only one possible coupling situation, namely case ③, for the coupling between EDCHF and microsphere. Figure 29b shows the schematic of the EDCHF based in-fiber resonator and corresponding diagram of light propagation [37].

**Fig. 30:**
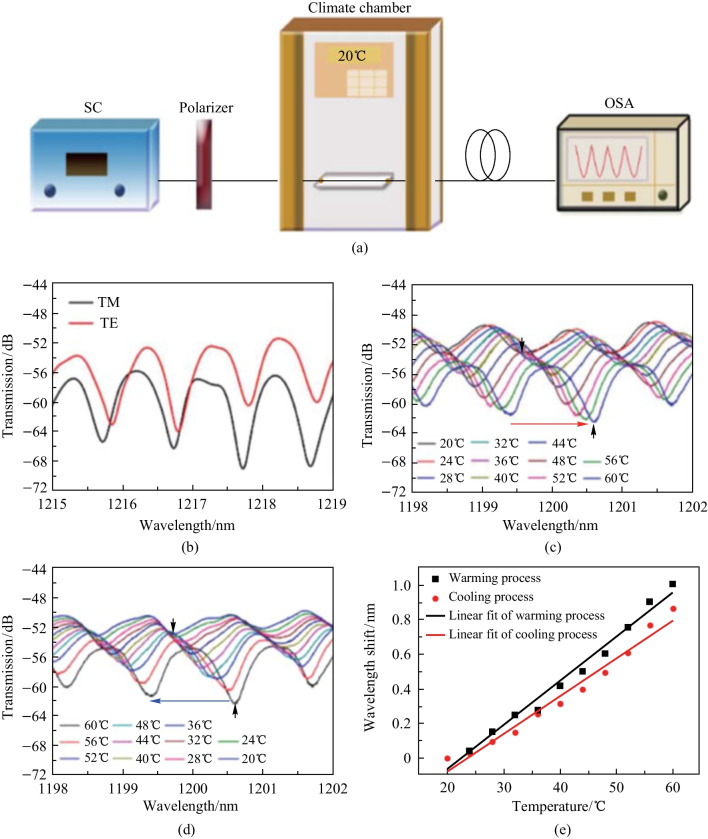
**a** Schematic of the experimental setup for researching the polarization and temperature sensing characteristics of the EDCHF based in-fiber resonator. **b** Spectrum evolution of warming process. **c** Spectrum evolution of cooling process. **d** Transmission vs wavelength. **e** Linear fit of wavelength shifts. Reproduced with permission from Ref. [37]

Unlike the in-fiber resonator based on SDCHF introduce previously, the in-fiber resonator based on EDCHF had an additional output port made of SMF. Thus the same fusion splicing and tapering processes was needed as for input port. Figure 30a shows the schematic of the setup for measuring the transmission spectra and for characterizing the temperature sensing properties of the device. The polarizer was added to vary the polarization state of the incident light. Their results indicate that the TE and TM modes of WGM have a small difference in the resonance wavelength, as shown in Fig. 30b. Then the temperature dependence of the in-fiber WGM microsphere resonator was then investigated and the results they obtained can be found in Fig. 30c–e. As temperature increases, the resonance wavelength has a red shift with a sensitivity of 25.5 pm/°C. On the other hand, as temperature decreases, the resonance wavelength has a blue shift with a sensitivity of 21.9 pm/°C [37].

### Multicore fiber facet based coupler

**Fig. 31:**
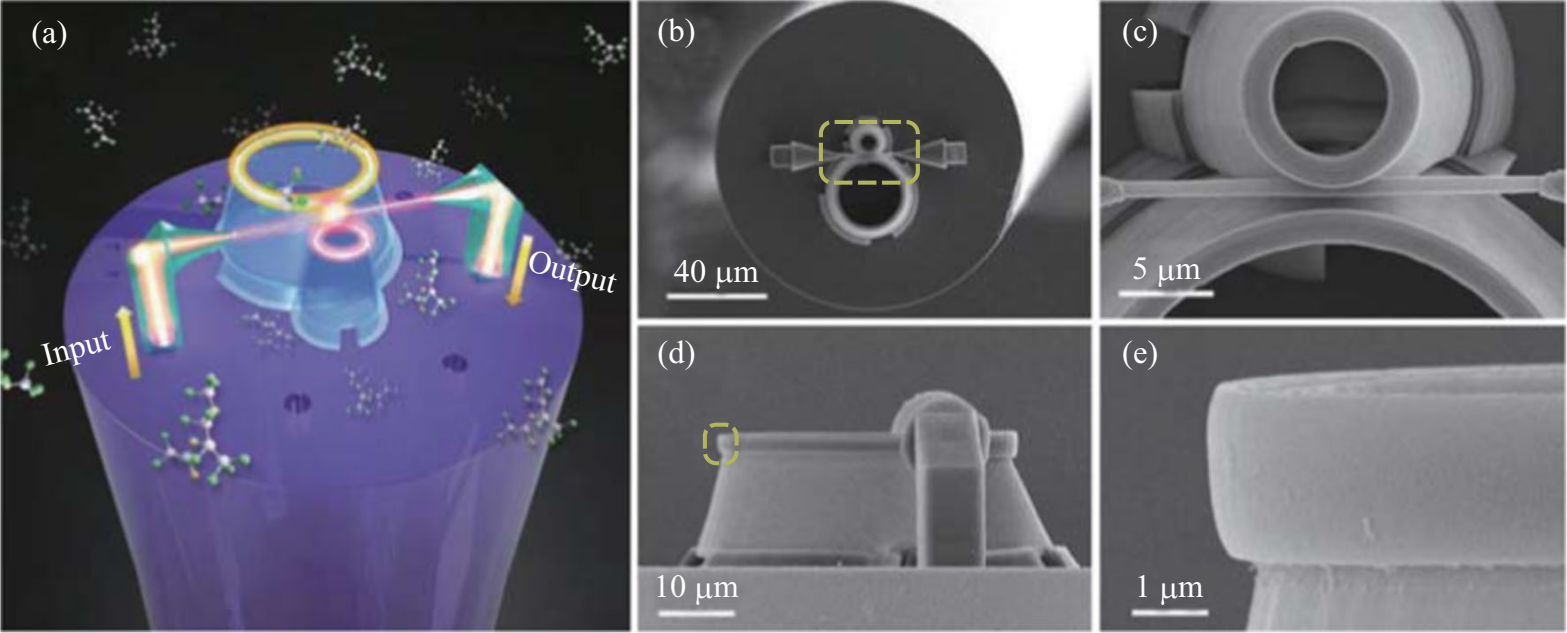
**a** Geometrical framework and optical path of the seven-core fiber facet based in-fiber resonator. Top view SEM images of the **b** whole and **c** part of the multicore fiber facet based in-fiber resonator. Side view SEM images of the **d** whole and **e** part of the multicore fiber facet based in-fiber resonator. Reproduced with permission from Ref. [81]

By virtue of the advanced 3D manufacturing technology of two-photon lithography, multicore fibers without hollow structure have the potential for constructing in-fiber resonator by fabricating an optical microsystem on the fiber facet. In 2019, Zhang et al. proposed facet based in-fiber resonator on a seven-core fiber [81], the structure and optical path are shown in Fig. 31a. Although the resonator is annular rather than spherical, the preparation technology and operating principle are the same if the microring is replaced by a microsphere, indicating the feasibility of a multicore fiber facet based in-fiber microsphere resonator. Such an in-fiber resonator can be also considered as the extension of suspended multicore hollow fiber based in-fiber microresonator with the original hollow structure replaced by the microsystem on facet.

Figure 31a shows that the microsystem on facet is composed of two micropillars, two prisms, two tapers, a waveguide and two ring resonators. The two micropillars stand on opposite cores of the seven-core optical fiber and support the prisms, guiding light out of the fiber or vice versa. The light are coupled to the waveguide via two tapers that are connected to the prisms, and excite the ring through evanescent wave coupling. Two hollow circular truncated cones with different sizes at opposite sides of the waveguide support the two rings. In fact, a WGM microsphere can be also embedded into the hollow truncated cones. To enhance the stability of the system, the waveguide was embedded into the gap between the smaller ring and corresponding hollow circular truncated cone, and the gap between the larger ring and the waveguide is controlled to realize critical coupling.

**Fig. 32:**
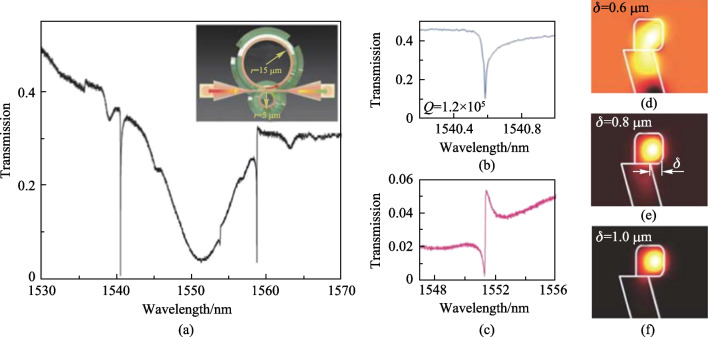
**a** Transmission spectrum of the device. Inset: the main structure. **b** Enlarged view of the resonant mode at wavelength of 1540.58 nm. **c** Typical antisymmetric Fano resonance around 1551.57 nm. **d**–**f** Electric field distribution of the resonant modes with *δ* of 0.6, 0.8, and 1 μm, respectively. Reproduced with permission from Ref. [81]

Zhang et al. fabricated this coupling structure by two-photon lithography [81]. The scanning electron microscopy (SEM) images are shown in Fig. 31b–d. Transmission properties were also tested and analyzed. Figure 32a shows the typical transmission spectrum of the device, with three significant dips in the wavelength range of 1530–1570 nm. It was found that the two narrow dips at wavelengths of 1540.58 and 1558.69 nm originated from the larger ring while the broad dip at 1551.35 nm was from the smaller ring. *Q* factor of 1.2 × 10^5^ was obtained at the dip of 1540.58 nm with details given in Fig. 32b, which is close to the maximum *Q* factor obtained so far in optical structures by 3D manufacturing. Moreover, another six small dips were observed in the spectrum, which were thought to be resulted from higher order modes. The one around 1551.57 nm was found to interfere with the broad dip, leading to a typical Fano line shape, as shown in Fig. 32c. It was also found that the protruding of the ring waveguide from the truncated cone, denoted by *δ*, had an important effect on light confinement of the ring. From Fig. 32d–f, it can be seen that the light is well confined in the ring when *δ* is equal to and larger than 0.8 μm.

**Fig. 33:**
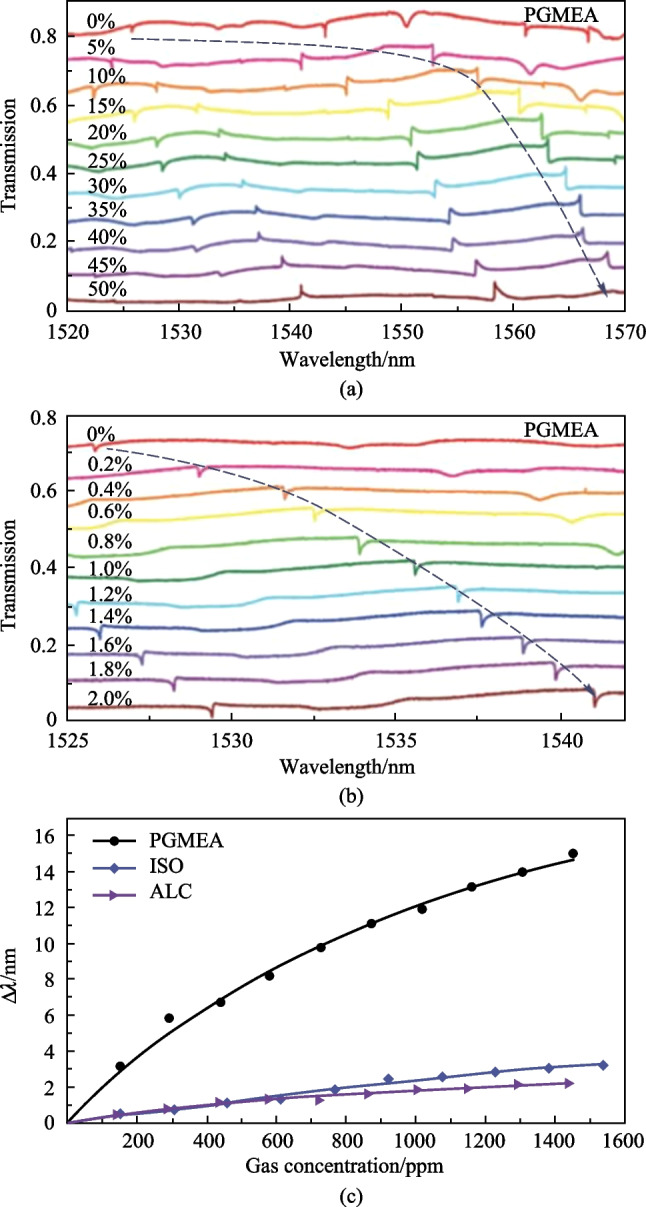
Spectrum evolutions when the device is placed in the vapor produced from PGMEA aqueous solution with concentration varying **a** from 0 to 50% and **b** from 0 to 2%. **c** Wavelength shift (Δ*λ*) dependence on gas concentration. Reproduced with permission from Ref. [81]

In such a device, the resonator as sensing element is exposed to the external environment. Hence, the device is suitable for gas sensing, which was experimentally demonstrated by the same authors, the results [81] are shown in Fig. 33. The gas was produced from the propylene glycol monomethyl ether acetate (PGMEA)-water liquid with different concentrations in a chamber. Figure 33a shows the evolution of the transmission spectrum of the device for PGMEA-water liquid’s concentration varying from 0 to 50%. It was found that resonance wavelengths redshifted with increasing concentration and the resonance wavelengths shifted faster in the concentration range of 0–10% than in the concentration range of 10%–50%. They attributed the reason to the fact that the PGMEA molecules are more easily adsorbed onto the fresh surface of the polymer ring. Figure 33b shows the spectrum evolution for concentrations varying from 0 to 2%, which further verifies the above analysis. Figure 33c plots the relationship between resonant wavelength shift of the device and the concentrations of PGMEA, isopropanol (ISO) and alcohol (ALC) vapors.

## In-fiber resonator with active microsphere

### Dye-doped microsphere locked onto MOF

**Fig. 34:**
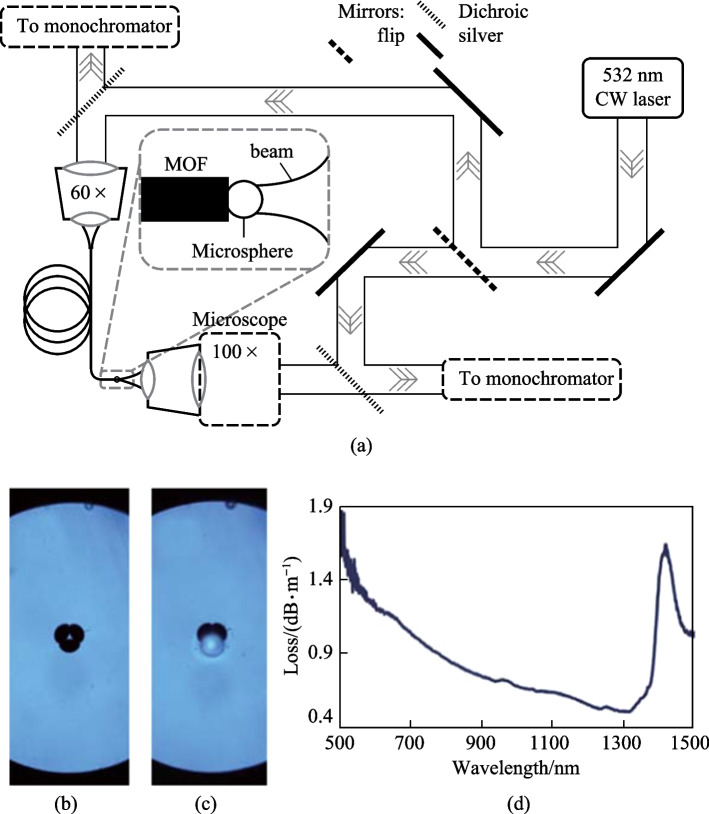
**a** Excitation and collection system for the WGMs in the in-fiber active microsphere. Microscopic images of a wagon wheel MOF **b** without and **c** with a dye-doped polystyrene sphere. **d** Optical loss of the MOF as a function of wavelength. Reproduced with permission from Ref. [24]

Compared with passive microspheres, active microspheres can be applied in a wider range of field including biosensing, optical thermometry based on fluorescence, lasing, etc. The earlier in-fiber coupling structures with an active microsphere utilized a hole on the tip of a MOF to hold the microsphere, as is the case for the typical example proposed by Francois et al. in 2011 [24], as shown in Fig. 31. The excitation and collection system for the WGMs in the in-fiber active microsphere is displayed in Fig. 34a. The active microsphere could be excited via a microscope (100× magnification objective), which could also be used to observe and record the emitted fluorescence signal. Alternatively, excitation and collection functions could be realized by the wagon wheel MOF, where the dye-doped polystyrene sphere was placed on the tip of a wagon wheel MOF and anchored in a hole. Figure 34b and c show the microscopic images of the MOF without and with the active microsphere, respectively. The typical loss spectrum of the MOF is shown in Fig. 34d, indicating the relatively low loss for visible light.

**Fig. 35:**
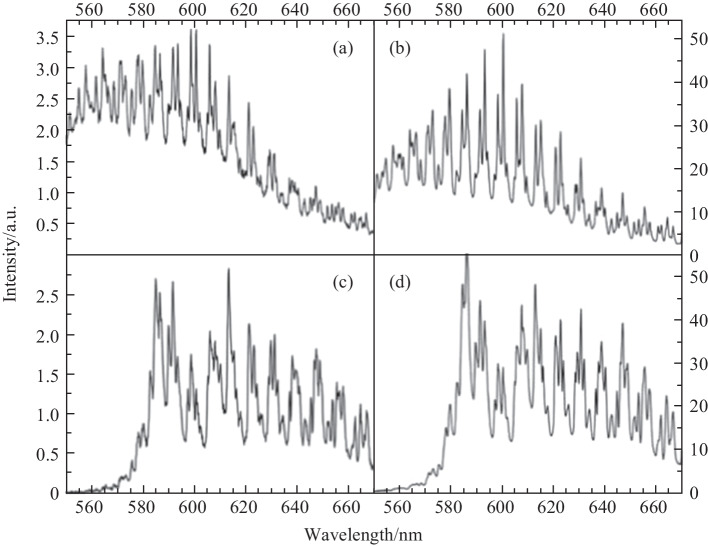
WGM spectra of a microsphere under different excitation and collection schemes: **a** microscope excitation and collection, **b** MOF excitation and microscope collection, **c** microscope excitation and MOF collection, and **d** MOF excitation and collection. Reproduced with permission from Ref. [24]

Figure 35 shows WGM-modulated fluorescence spectra of the in-fiber active microsphere with four different configurations of excitation and collection. Comparing Fig. 35a and b where microscope collection was adopted, it was found that the intensity of the fluorescence signal was significantly higher (~ 9.2 fold increase) when the MOF rather than the microscope was used for excitation. When the MOF was used for collection, the collected fluorescence intensity with MOF excitation was much higher (~ 19 fold increase), as shown in Fig. 35c and d. The higher fluorescence intensity with MOF excitation was attributed to the higher overlap of the excitation beam and the microsphere in this case, where guided light in MOF was strongly confined in the small suspended core with diameter of 1.5 μm.

**Fig. 36:**
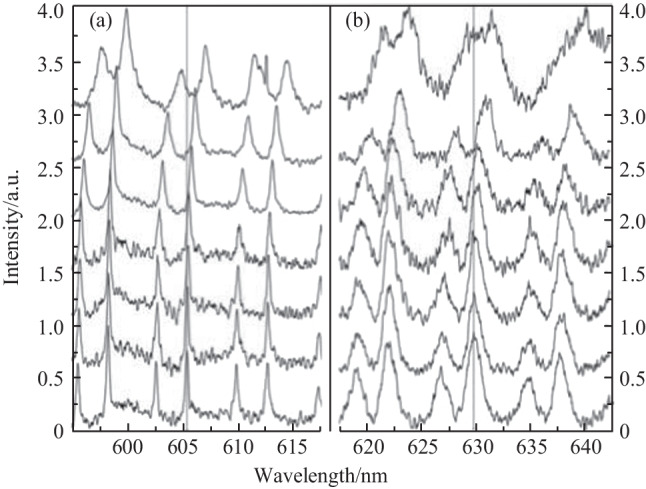
WGM spectrum evolution of a microsphere embedded within **a** the microfluidic flow cell and **b** the MOF with increasing glycerol concentration (bottom to top: DI water, 0.78125%, 1.5625%, 3.125%, 6.25%, 12.5%, 25%). Reproduced with permission from Ref. [24]

The above results prove that excitation efficiency for the MOF based in-fiber active microsphere can be significant increased. In addition, the MOF based in-fiber active microsphere can work as a probe, which significantly simplifies the sensing architecture. When the microsphere was dipped into water/glycerol solutions of increasing glycerol concentration, Fig. 36a and b show the WGM spectrum evolutions of the same batch of active microspheres embedded in the microfluidic flow cell and the MOF, respectively. It was found that the microspheres exhibited similar sensitivities of 56.9 and 45.5 nm/RIU, which implied that locking the active microsphere onto the tip of MOF did not change the sensing performance significantly. This in-fiber coupling structure can be also used for label-free biosensing if the microsphere is surface-functionalized.

**Fig. 37:**
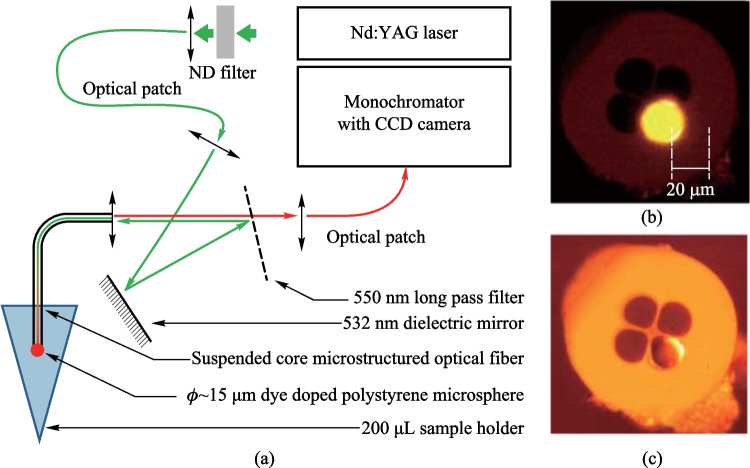
**a** Schematic of the in-vivo biological sensing platform. **b** Bright-field and **c** fluorescence images of an active microsphere anchored onto the tip of MOF. Reproduced with permission from Ref. [28]

**Fig. 38:**
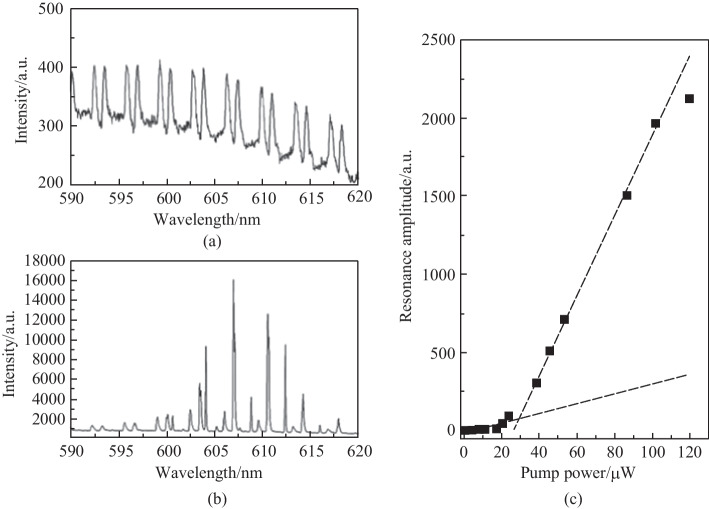
Spectra **a** below and **b** above threshold. **c** Resonance amplitude as a function of pump power. Reproduced with permission from Ref. [28]

In 2015, François et al. designed an *in-vivo* biological sensing platform [28], where the excitation and collection functions were both realized by the MOF. The probe composed of a biotin functionalized dye-doped polystyrene microsphere anchored onto the tip of MOF was dipped into the 200 μL sample. Figure 37b and c show the bright-field and fluorescence images of the probe, respectively. Lasing behavior of the dye doped active microsphere was characterized, as shown in Fig. 38. The spectra of the active microsphere below and above the lasing threshold are shown in Fig. 38a and b, respectively, and the threshold calculated from Fig. 38c is 28 μW. It was found that the lasing modes had a higher *Q* factor of 1.5 × 10^4^ compared with the fluorescent modes with *Q* factor of 3 × 10^3^. The increase of *Q* factor is highly beneficial for sensing purposes as it increases the resolution of the sensor.

**Fig. 39:**
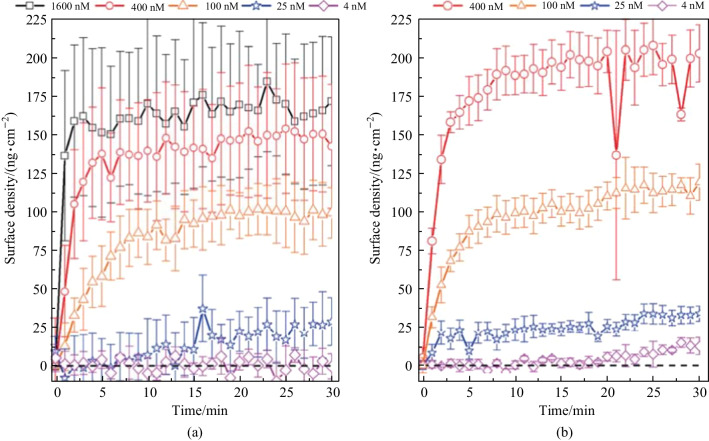
Binding kinetic for neutravidin on a biotin-functionalized active microsphere operating **a** below and **b** above the lasing threshold. Reproduced with permission from Ref. [28]

The Binding kinetic for neutravidin on a functionalized active microsphere with biotin-D was then analyzed [28], for the operation modes below and above the lasing threshold respectively, as shown in Fig. 39a and b, When operating below threshold, It was found that the highest concentration in the experiment (1600 nM) could be easily detected. Moreover, the saturation of radius increase of the microsphere was achieved within the first minutes and the saturation for lower concentrations was achieved later. In contrast, in the case of operating above threshold, the saturation of the functionalized sensor surface upon exposure to the 400 nM neutravidin solution was achieved after few minutes and the other concentrations never achieved saturation within the time frame of 30 min. This eventually led to the experimental [28] result that, for the smallest concentration of 4 nM, a slight wavelength shift could be observed with the lasing WGM, but no wavelength shift could be observed with the fluorescent WGM. The result is quite reasonable considering the significant increase of the *Q* factor of the lasing WGM and the subsequent enhancement of detection limit.

### Rare-earth ion doped microsphere embedded into micro-structure hollow fiber

**Fig. 40:**
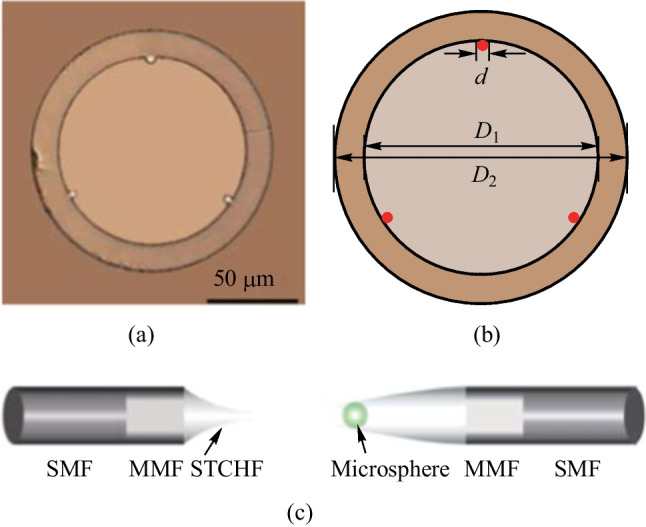
**a** Microscopic image and **b** schematic of the cross section of the STCHF. **c** Schematic of the in-fiber coupling structure with STCHF and coupled active microsphere. Reproduced with permission from Ref. [42]

The dye doped polymer microspheres have the disadvantage of photobleaching, whereas rare-earth ion doped glass microspheres do not. Moreover, compared with the MOF based in-fiber active microcavity where the microsphere is anchored on the tip, the microsphere can be embedded into the cone region produced by half-collapse of the cladding for the micro-structure hollow fiber, and this significantly enhances the stability of the coupling structure. Figure 40a and b show the microscopic image and schematic of the cross section of a suspended tri-core hollow fiber (STCHF) [42], respectively. Due to the asymmetry of the three suspended cores, the coupling of microsphere and STCHF is similar to the case ④, as shown in Fig. 27a, where the pump light is injected into the microsphere by evanescent wave coupling. In 2019, Zhang et al. proposed a STCHF based in-fiber coupling structure, which was composed of SMF, MMF, STCHF, and microsphere [42], as shown in Fig. 40c.

**Fig. 41:**
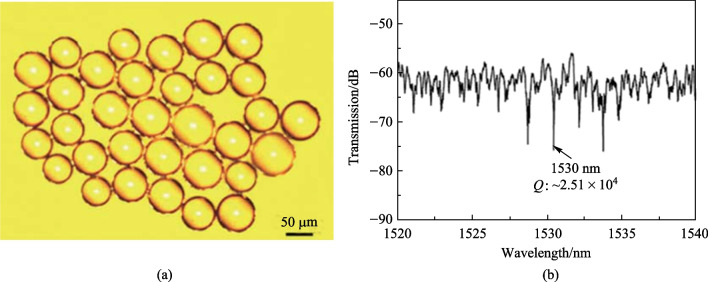
**a** Micrograph and **b** transmission spectrum of the Er^3+^-Yb^3+^ co-doped tellurite glass microsphere. Reproduced with permission from Ref. [42]

In Ref. [42], the active microsphere adopted was the Er^3+^-Yb^3+^ co-doped tellurite glass microsphere with material component of 72TeO_2_-20ZnO-5Na_2_CO_3_-1.5Y_2_O_3_-0.5Er_2_O_3_-1Yb_2_O_3_ [42]. The microsphere was fabricated by droplet sphere-forming method in three steps. First, the glass sample was prepared by melt-quenching method. Second, the prepared glass sample was ground into powder and passed through a 300 mesh sieve. Finally, the fine powders were dropped through a vertical furnace and melted into microspheres, which were cooled and solidified, as shown in Fig. 41a. The transmission spectrum of the structure shown in Fig. 40c with the microsphere coupled with the fiber taper was found to be a typical WGM spectrum, as shown in Fig. 41b.

**Fig. 42:**
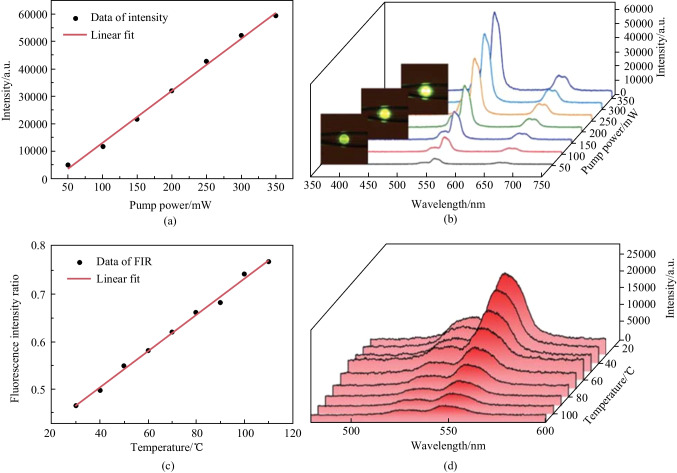
**a** Experimental data with linear fit and **b** spectrum evolution of the luminescence intensity of the microsphere coupled with STCHF as pump power increases. **c** Experimental data with linear fit and **d** spectrum evolution of the luminescence intensity of the microsphere coupled with STCHF as temperature increases. Reproduced with permission from Ref. [42]

As the pump power increased, the luminescence intensity of the microsphere coupled with STCHF also increased, as shown in Fig. 42a and b. In addition, as the temperature increased from 303 to 383 K in steps of 20 K, the intensity ratio of the peak around 528 nm to the peak around 549 nm also increased, which was thought to be originated from the thermally coupled levels of ^2^H_11/2_ and ^4^S_3/2_ in Er^3+^, as shown in Fig. 42c and d.

**Fig. 43:**
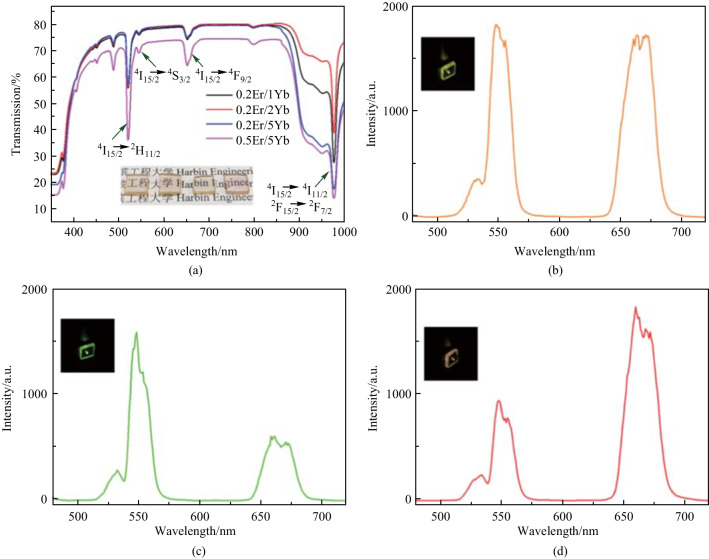
**a** Absorption spectrum of the Er^3+^-Yb^3+^ co-doped lead germanate glass. The luminescence spectra of **b** PGG1, **c** PGG2 and **d** PGG3 glass samples. Inset: images of the three glass samples under luminescence. Reproduced with permission from Ref. [46]

Color variation of the Er^3+^-Yb^3+^ co-doped lead germanate glass microsphere was also investigated [46]. Glass samples were prepared by the above-mentioned melt-quenching method and the chemical compositions of the host media were 45 mol % PbO, 45 mol % GeO_2_, and 10 mol % Ga_2_O_3_. Four different proportions of Er^3+^ and Yb^3+^ were considered and their chemical compositions denoted by (*x*, *y*) were equal to (0.2, 1), (0.2, 2), (0.2, 5) and (0.5, 5) for the four kinds of glass samples named by PGG1, PGG2, PGG3 and PGG4, respectively. Figure 43a shows the absorption spectrum of the glass samples with different proportions of Er^3+^ and Yb^3+^. It was found that three absorption peaks existed at 524, 547, and 659 nm in the visible range, derived from the transitions of Er^3+^ from ^4^I_15/2_ to ^2^H_11/2_, ^4^S_3/2_ and ^4^F_9/2_, respectively.

The luminescence spectra of PGG1, PGG2 and PGG3 glass samples under the same optical pumping are shown in Figs. 43b–d. As the two peaks around 547 and 659 nm are dominant while the peak around 524 nm is weak, the former two peaks was mainly considered in ref. [46]. The intensity ratio of the red emission around 659 nm to the green emission around 547 nm was found to be different for glass samples with different Er^3+^ /Yb^3+^ concentration ratios, and this difference induced variations of the luminescence color, as shown in the insets of Fig. 43b–d. The color variation was attributed to both the energy transfer process between Er^3+^ and Yb^3+^ and the cross relaxation process between Er^3+^ ions. When the intensities of the red and green emissions were similar, the luminescence color of the microsphere was yellow, as shown in Fig. 43b. In contrast, the luminescence color was mainly determined by the dominant emission if the emission intensities were greatly different, as shown in Fig. 43c and d.

**Fig. 44:**
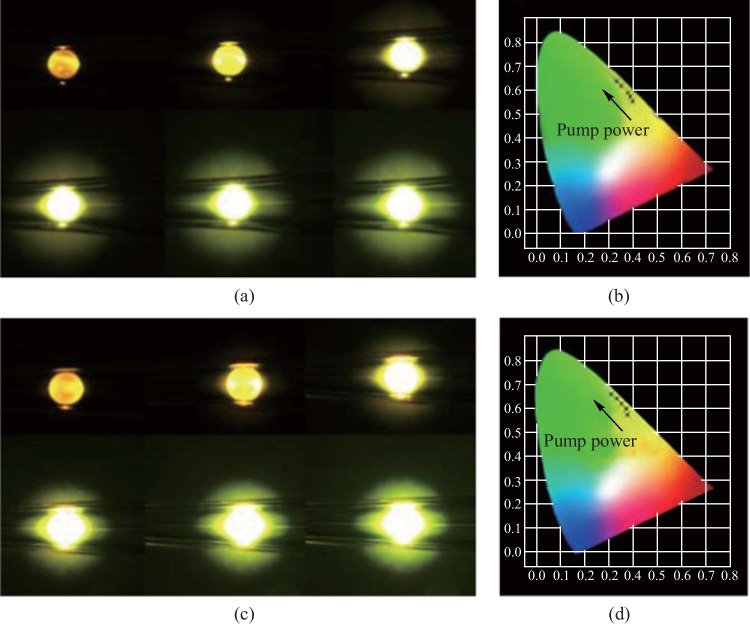
Microscopic images of **a** PGG3 and **c** PGG4 glass microspheres with increased pump power. CIE chromaticity diagrams of **b** PGG3 and **d** PGG4 glass microspheres for different pump powers. Reproduced with permission from Ref. [46]

In addition to the Er^3+^/Yb^3+^ concentration ratio, it was found that the pump power could readily tune the luminescence color. When the pump power was low, the intensities of the red and green emissions were similar and the luminescence color was yellow. With the increase of the pump power, the green emission intensity was found to increase and then the luminescence color changed from yellow to green, as shown in Fig. 44a and c. CIE chromaticity diagram was a useful way to visualize the color change more accurately. The corresponding CIE chromaticity diagrams of the color changes shown in Fig. 44a and c were obtained, as shown in Fig. 44b and d. It is found that as the pump power increases, the luminescence color is closer to the pure green. This characteristic provides the in-fiber Er^3+^/Yb^3+^ co-doped lead germanate glass microsphere with the potential to be applied in multicolor displays.

## Discussion

In-fiber resonators have the features of compact structure and high stability, which have become a new sub-field of WGM photonic devices [56–58, 60, 61, 82, 83]. In terms of application, the self-alignment makes the in-fiber resonator based on WGM stable, and thus practical especially for sensing. Moreover, the in-fiber resonator can work as a convenient sensing probe due to its reflection mode operation. Heretofore, it has been used in the sensing of temperature [50, 53], humidity [84], hydrostatic pressure [78], refractive index [39], chemical vapor [81], biomolecule identity [28, 85], etc. The sensing functions are realized by monitoring the change of WGM resonance. Furthermore, the spectral peak or dip of WGM resonance makes in-fiber resonator capable of optical filtering [39], where the peak and dip are found in the operation mode of reflection and transmission, respectively. Meanwhile, the WGM peak can also be regarded as a narrow-band wavelength-selective reflector [57]. In addition, due to the diverse and flexible structures of the in-fiber resonator, a variety of optical mechanisms such as EIT [53] and Fano resonance [49] can be utilized to improve the properties or functionalities of photonic devices, such as optimize optical filtering [55] and realize optical switching [54]. The adoption of an active microsphere in the in-fiber resonator breaks the application limitation of the passive microsphere and further expands the application fields, including tunable luminescence colors [46], laser [28, 48], self-reference biosensing [47], etc.

However, realizing high coupling efficiency for in-fiber resonator remains more challenging than for other coupling schemes. As such, it is critical to design optical fibers with optimized microstructures and optical microsystems for high-efficiency coupling [86–90]. In addition, the multi-resonator based in-fiber resonator is a promising platform to study certain optical phenomena with profound physical significance [84, 91], such as mode splitting [68], Vernier effect [92] and parity-time (PT) symmetry [93]. Specifically, mode splitting has been observed and utilized for displacement and temperature sensing with improved *Q* factor [40, 94]. The Vernier effect can be applied for weak signal detection thanks to a sensitivity amplification effect [95] and can be also utilized to produce single wavelength lasing [96]. Meanwhile, by exploiting the breaking of PT symmetry, single-wavelength operation can be established for lasers as well [93]. Moreover, sensitivity enhancement can be achieved based on the exceptional points associated with PT symmetry breaking, because the eigenvalue splitting shows direct dependence on *ε*^1/*N*^ instead of being proportional to the perturbation strength *ε* [97]. Here, *N* is the order of exceptional point at which *N* eigenvalues and the corresponding eigenvectors coalesce.

Microsphere material is another important area of research. Materials that are sensitive to specific parameters are necessary for high-sensitivity sensing [98]. For instance, materials with higher thermo-expansion and thermo-optic coefficients are perferred for the ultrahigh temperature sensitivity [99]. The microstructure of material also plays a significant role in applications, such as the porous glass microsphere (PGM) for chemical vapor sensing [27]. The pores distributed in the microsphere can adsorb chemical vapor molecules, leading to a refractive index change of the PGM and thus the resonance wavelength shift of the WGMs. Finally, research on the dopant and matrix materials is vital to bio-chemical sensing [24, 25], optical thermometry [42], specific wavelength lasing [100], nonlinear optics [101], etc. In brief, the research on the in-fiber WGM microsphere coupler is continuing, and there are a lot of unexplored areas for emerging application of WGM microsphere couplers, which will promote the development of photonic devices.

## Conclusion

We have reviewed recent progress of in-fiber WGM microsphere resonators consisting of optical fiber based in-fiber coupler and microsphere, and introduced typical instances for the coupling structures. The conventional fiber can be used to fabricate the in-fiber coupler and there are two schemes to produce space to accommodate the microsphere. One is inscribing a microstructure on the D-shaped fiber by laser ablation [15]. Another is forming a cone in the fiber by laser ablation and chemical etching [59], which has a better stability because the microsphere can be locked firmly into the cone. The capillary with air core can simplify the fabrication process, with the conical structure divided as cone-shaped inwall capillary by internally etching [49] and half collapsed capillary by arc discharge [26]. The FLMT can be employed to inscribe a rectangle notch on the etched capillary for reducing coupling paths and increasing *Q* factor [52]. The half-collapsed-capillary based in-fiber coupler needs neither etching nor FLMT, and involves both evanescent wave coupling and free space coupling [77]. Furthermore, when the solid microsphere inside capillary is replaced by a HGM, the two-beam interference can be realized [78]. Besides the capillary, micro-structure hollow fibers can be also employed to construct in-fiber couplers, including the HACF [50], SDCHF [39] and EDCHF [37]. High coupling efficiencies can be achieved by optimizing the cone-apex angle of coupler. The multicore fiber facet based coupler is an important development direction in the future as an extension of the SDCHF. In addition to introducing each coupling structure and corresponding principle, we also introduced their potential applications. Moreover, active microspheres, including dye doped polymer microsphere [24] and rare-earth ion doped glass microsphere [42] can be used to broaden the application range of the in-fiber resonators. Biosensing, lasing, optical thermometry and color variation based on active microspheres have been comprehensively demonstrated. At the end of this review, we analyzed the current status of in-fiber WGM microsphere resonator and suggested a lot of unexplored areas for research, including structure optimization, multi-resonator coupler platform and resonator material engineering. The in-fiber WGM microsphere resonator has advantages of alignment-free, reflection mode operation and compact structure, offering great potential in photonic integration.

## References

[1] Vahala KJ (2003). Optical microcavities. Nature.

[2] Ding Y, Fan H, Zhang X, Jiang X, Xiao M (2017). Ultralow-threshold neodymium-doped microsphere lasers on a silicon chip. Opt. Commun..

[3] Jiang X, Xiao Y, Zou C, He L, Dong C, Li B, Li Y, Sun FW, Yang L, Gong Q (2012). Highly unidirectional emission and ultralow-threshold lasing from on-chip ultrahigh-*Q* microcavities. Adv. Mater..

[4] Wei C, Gao M, Hu F, Yao J, Zhao YS (2016). Excimer emission in self-assembled organic spherical microstructures: an effective approach to wavelength switchable microlasers. Adv. Opt. Mater..

[5] Vollmer F, Yang L (2012). Review Label-free detection with high-*Q* microcavities: a review of biosensing mechanisms for integrated devices. Nanophotonics.

[6] Lee A, Lee SK, Sang HL (2019). Simultaneous temperature and strain sensing with hybrid resonator of fiber Bragg grating and whispering gallery resonator. IEEE Sens. J..

[7] Arnold S, Khoshsima M, Teraoka I, Holler S, Vollmer F (2003). Shift of whispering-gallery modes in microspheres by protein adsorption. Opt. Lett..

[8] Xue X, Zheng X, Weiner A (2017). Soliton trapping and comb self-referencing in a single microresonator with χ(2) and χ(3) nonlinearities. Opt. Lett..

[9] Rokhsari H, Kippenberg TJ, Carmon T, Vahala KJ (2006). Theoretical and experimental study of radiation pressure-induced mechanical oscillations (parametric instability) in optical microcavities. IEEE J. Sel. Top. Quantum Electron..

[10] Schliesser A, Kippenberg TJ (2010). Cavity optomechanics with whispering-gallery-mode optical micro-resonators. Adv. At. Mol. Opt. Phys..

[11] Cai X, Strain M, Yu S, Sorel M (2016). Photonic integrated devices for exploiting the orbital angular momentum of light in optical communications. Front Optoelectron..

[12] Vogt D, Jones A, Schwefel H, Leonhardt R (2018). Prism coupling of high-*Q* terahertz whispering-gallery-modes over two octaves from 0.2 THz to 1.1 THz. Opt. Express.

[13] Gorodetsky ML, Ilchenko VS (1998). Optical microsphere resonators: optimal coupling to high-*Q* whispering gallery modes. J. Opt. Soc. Am. B.

[14] Gorodetsky ML, Ilchenko VS (1994). High-*Q* optical whispering-gallery microresonators: precession approach for spherical mode analysis and emission patterns with prism couplers. Opt. Commun..

[15] Shi L, Zhu T, Huang D, Liang C, Liu M, Liang S (2017). In-fiber Mach-Zehnder interferometer and sphere whispering gallery mode resonator coupling structure. Opt. Lett..

[16] Shi L, Zhu T, Huang D, Liu M, Deng M, Huang W (2015). In-fiber whispering-gallery-mode resonator fabricated by femtosecond laser micromachining. Opt. Lett..

[17] Dubreuil N, Knight J, Leventhal DK, Sandoghdar V, Hare J, Lefèvre V (1995). Eroded monomode optical fiber for whispering-gallery mode excitation in fused-silica microspheres. Opt. Lett..

[18] Dong R, Fan Z, Liao J, Qavi A, Long GL, Yang L (2020). Highly efficient optical add-drop filter with an angle-polished fiber coupler. IEEE Photonics Technol. Lett..

[19] Ilchenko VS, Yao XS, Maleki L (1999). Pigtailing the high-*Q* microsphere cavity? A simple fiber coupler for optical whispering-gallery modes. Opt. Lett..

[20] Liu X, Zhang X, Zhang Q, Yang Y, Xie Y, Wang Z, Sun H, Huang Y, Wang T (2022). Trapezoidal angled fiber coupler for microsphere resonator with enhancement of Fano resonance. IEEE Photonics Technol. Lett..

[21] Hou F, Zhang X, Wang Z, Yang L, Sun W, Yang Y, Dong Y, Huang Y, Wang T (2020). Magnetic fluid infiltrated microbottle resonator sensor with axial confined mode. IEEE Photonics J..

[22] Yang D, Wang A, Chen JH, Yu XC, Lan C, Ji Y, Xiao Y (2020). Real-time monitoring of hydrogel gel-sol transition in an ultrahigh-*Q* microbubble resonator. Photon. Res..

[23] Yin Y, Niu Y, Ren M, Wu W, Zhao W, Nan J, Zheng Z, Zhang Y, Ding M (2018). Strain sensing based on a microbottle resonator with cleaned-up spectrum. Opt. Lett..

[24] Francois A, Rowland KJ, Monro TM (2011). Highly efficient excitation and detection of whispering gallery modes in a dye-doped microsphere using a microstructured optical fiber. Appl. Phys. Lett..

[25] Franois A, Rowland KJ, Afshar SV, Henderson MR, Monro TM (2013). Enhancing the radiation efficiency of dye doped whispering gallery mode microresonators. Opt. Express.

[26] Wang H, Lan X, Huang J, Yuan L, Kim CW, Xiao H (2013). Fiber pigtailed thin wall capillary coupler for excitation of microsphere WGM resonator. Opt. Express.

[27] Wang H, Yuan L, Kim CW, Lan X, Huang J, Ma Y, Xiao H (2015). Integrated chemical vapor sensor based on thin wall capillary coupled porous glass microsphere optical resonator. Sens. Actuators B Chem..

[28] François A, Reynolds T, Monro TM (2015). A fiber-tip label-free biological sensing platform: a practical approach toward *in-vivo* sensing. Sensors (Basel).

[29] Liu N, Shi L, Zhu S, Xu X, Yuan S, Zhang X (2018). Whispering gallery modes in a single silica microparticle attached to an optical microfiber and their application for highly sensitive displacement sensing. Opt. Express.

[30] Shi L, Tao Z, Huang D, Min L (2016). Electrical thermo-optic tuning of integrated polymethyl methacrylate sphere whispering gallery mode resonator. IEEE Photonics J..

[31] Wang R, Fraser M, Li J, Qiao X, Wang A (2015). Integrated in-fiber coupler for microsphere whispering-gallery modes resonator excitation. Opt. Lett..

[32] Schartner EP, Dowler A, Ebendorff-Heidepriem H (2017). Fabrication of low-loss, small-core exposed core microstructured optical fibers. Opt. Mater. Express.

[33] Liu X, Cui XL, Wang DN (2020). Integrated in-fiber coupler for a whispering-gallery mode microsphere resonator. Opt. Lett..

[34] Peng L, Riesen N, Li J, Han M, Nguyen LV, Ebendorff-Heidepriem H, Warren-Smith SC (2021). Whispering gallery mode excitation using exposed-core fiber. Opt. Express.

[35] Warren-Smith SC, Ebendorff-Heidepriem H, Foo TC, Moore R, Davis C, Monro TM (2009). Exposed-core microstructured optical fibers for real-time fluorescence sensing. Opt. Express.

[36] Li X, Wang D (2021). A whispering-gallery mode microsphere resonator based on optical fiber with an open microcavity. J. Lightwave Technol..

[37] Zhang M, Yang W, Tian K, Yu J, Li A, Wang S, Lewis E, Farrell G, Yuan L, Wang P (2018). In-fiber whispering-gallery mode microsphere resonator-based integrated device. Opt. Lett..

[38] Zhou A, Qin B, Zhu Z, Zhang Y, Liu Z, Yang J, Yuan L (2014). Hybrid structured fiber-optic Fabry-Pérot interferometer for simultaneous measurement of strain and temperature. Opt. Lett..

[39] Yang L, Zhang X, Yang Y, Hou F, Sun W, Sun H, Yu Q, Lian Z, Wang T (2020). All-pass and add-drop microsphere resonator in a suspended dual-core hollow fiber. IEEE Photonics Technol. Lett..

[40] Kosma K, Schuster K, Kobelke J, Pissadakis S (2018). An “in-fiber” whispering-gallery-mode bi-sphere resonator, sensitive to nanometric displacements. Appl. Phys. B.

[41] Ruan Y, Boyd K, Ji H, Francois A, Ebendorff-Heidepriem H, Munch J, Monro TM (2014). Tellurite microspheres for nanoparticle sensing and novel light sources. Opt. Express.

[42] Zhang M, Li A, Yu J, Lu X, Wang P, Lewis E, Farrell G, Yuan L, Wang P (2019). In-fiber temperature sensor based on green up-conversion luminescence in an Er^3+^-Yb^3+^ co-doped tellurite glass microsphere. Opt. Lett..

[43] Huang X, Zhou Y, Woo CM, Pan Y, Nie L, Lai P (2020). Multifunctional layered black phosphorene-based nanoplatform for disease diagnosis and treatment: a review. Front Optoelectron..

[44] Savelieva TA, Kuryanova MN, Akhlyustina EV, Linkov KG, Meerovich GA, Loschenov VB (2020). Attenuation correction technique for fluorescence analysis of biological tissues with significantly different optical properties. Front Optoelectron..

[45] Yakovlev D, Farrakhova D, Shiryaev A, Efendiev K, Loschenov V (2021). New approaches to diagnostics and treatment of cholangiocellular cancer based on photonics methods. Front Optoelectron..

[46] Zhang M, Tian K, Wang S, Yuan L, Farrell G, Lewis E, Wang P (2020). Color variation of the up-conversion luminescence in Er^3+^-Yb^3+^co-doped lead germanate glasses and microsphere integrated devices. J. Lightwave Technol..

[47] Reynolds T, Franois A, Riesen N, Turvey ME, Nicholls SJ, Hoffmann P, Monro TM (2016). Dynamic self-referencing approach to whispering gallery mode biosensing and its application to measurement within undiluted serum. Anal. Chem..

[48] Li A, Tian K, Yu J, Minz RA, Ward JM, Mondal S, Wang P, Chormaic SN (2021). Packaged whispering gallery resonator device based on an optical nanoantenna coupler. Opt. Express.

[49] Zhang X, Yang Y, Shao H, Bai H, Pang F, Xiao H, Wang T (2017). Fano resonances in cone-shaped inwall capillary based microsphere resonator. Opt. Express.

[50] Wang J, Zhang X, Yan M, Yang L, Hou F, Sun W, Zhang X, Yuan L, Xiao H, Wang T (2018). Embedded whispering-gallery mode microsphere resonator in a tapered hollow annular core fiber. Photon. Res..

[51] Bai X, Wang D (2018). Whispering-gallery-mode excitation in a microsphere by use of an etched cavity on a multimode fiber end. Opt. Lett..

[52] Zhang X, Bai H, Pan H, Wang J, Yan M, Xiao H, Wang T (2018). In-line fiber Michelson interferometer for enhancing the *Q* factor of cone-shaped inwall capillary coupled resonators. IEEE Photonics J..

[53] Zhang X, Yang Y, Bai H, Wang J, Yan M, Xiao H, Wang T (2017). Theoretical aspects and sensing demonstrations of cone-shaped inwall capillary-based microsphere resonators. Photon. Res..

[54] Wu JH, Gao JY, Xu JH, Silvestri L, Artoni M, La Rocca G, Bassani F (2005). Ultrafast all optical switching via tunable Fano interference. Phys. Rev. Lett..

[55] Yu, H., Li, Y., Yu, H., Chen, M., Hoekman, M., Chen, H., Leinse, A., Heideman, R.G., Mateman, R., Yang, S., Xie, S.: Record high-*Q* optical bandpass filter based on the EIT-like effect between two microrings. In: Proceedings of Optical Fiber Communication Conference, OSA Technical Digest (online) (Optica Publishing Group), Th1K.5. (2016)

[56] Shi L, Gao Q, Wang Q, Jiang L, Luo J, Zhu T (2022). Two-dimensional tapered optical fiber core for whispering gallery mode excitation. IEEE Photonics Technol. Lett..

[57] Shi L, Wang Q, Luo J, Zhu T (2021). In-fiber wavelength-selective reflector based on Y-junction coupled whispering gallery mode resonator. Opt. Lasers Eng..

[58] Bai X, Wang D (2020). An in-fiber coupler for whispering-gallery-mode excitation in a microsphere resonator. IEEE Photonics Technol. Lett..

[59] Li J, Wang D, Yan J, Ge Y (2022). A reflective whispering gallery mode microsphere resonator in single-mode fiber. IEEE Photonics Technol. Lett..

[60] Hua K, Wang D (2021). A whispering gallery mode microsphere resonator integrated with angle polished multimode fiber. J. Lightwave Technol..

[61] Özel B, Nett R, Weigel T, Schweiger G, Ostendorf A (2010). Temperature sensing by using whispering gallery modes with hollow core fibers. Meas. Sci. Technol..

[62] Semouchkina E, Duan R, Semouchkin G, Pandey R (2015). Sensing based on Fano-type resonance response of all-dielectric metamaterials. Sensors.

[63] Liao J, Wu X, Liu L, Xu L (2016). Fano resonance and improved sensing performance in a spectral-simplified optofluidic micro-bubble resonator by introducing selective modal losses. Opt. Express.

[64] Asano M, Oezdemir SK, Chen W, Ikuta R, Yang L, Imoto N, Yamamoto T (2016). Controlling slow and fast light and dynamic pulse-splitting with tunable optical gain in a whispering-gallery-mode microcavity. Appl. Phys. Lett..

[65] Mingaleev SF, Miroshnichenko AE, Kivshar YS (2008). Coupled-resonator-induced reflection in photonic-crystal waveguide structures. Opt. Express.

[66] Yao Y, Cheng Z, Dong J, Zhang X (2020). Performance of integrated optical switches based on 2D materials and beyond. Front Optoelectron..

[67] Nozaki K, Shinya A, Matsuo S, Sato T, Kuramochi E, Notomi M (2013). Ultralow-energy and high-contrast all-optical switch involving Fano resonance based on coupled photonic crystal nanocavities. Opt. Express.

[68] Peng B, Ozdemir S, Chen W, Nori F, Yang L (2014). What is and what is not electromagnetically-induced-transparency in whispering-gallery-microcavities. Nat. Commun..

[69] Xiao Y, Li M, Liu Y, Li Y, Sun X, Gong Q (2010). Asymmetric Fano resonance analysis in indirectly coupled microresonators. Phys. Rev. A.

[70] Zheng C, Jiang X, Hua S, Chang L, Li G, Fan H, Xiao M (2012). Controllable optical analog to electromagnetically induced transparency in coupled high-*Q* microtoroid cavities. Opt. Express.

[71] Shang Y, Ye Y, Lin X (2017). Experimental observation of Fano-like resonance in a whispering-gallery-mode microresonator in aqueous environment. Photon. Res..

[72] Miao Y, Peng Y, Xiang Y, Li M, Lu Y, Song Y (2016). Dynamic Fano resonance in thin fiber taper coupled cylindrical microcavity. IEEE Photonics J..

[73] Wei D (2013). Fano resonances in metallic grating coupled whispering gallery mode resonator. Appl. Phys. Lett..

[74] Zou C, Shu F, Sun F, Gong Z, Han Z, Guo G (2013). Theory of free space coupling to high-*Q* whispering gallery modes. Opt. Express.

[75] Siegle T, Kellerer J, Bonenberger M, Krämmer S, Klusmann C, Müller M, Kalt H (2018). Comparison of various excitation and detection schemes for dye-doped polymeric whispering gallery mode micro-lasers. Opt. Express.

[76] Zhu J, Ozdemir S, Yilmaz KH, Peng B, Dong M, Tomes M, Carmon T, Yang L (2014). Interfacing whispering-gallery microresonators and free space light with cavity enhanced Rayleigh scattering. Sci. Rep..

[77] Yang Y, Zhang X, Liu X, Wang Z, Yu Y, Wang J, Wang T (2020). In-fiber zigzag excitation for whispering-gallery modes via evanescent wave and free space coupling. Opt. Express.

[78] Sun H, Zhang X, Liu X, Wang Z, Yu Y, Yang Y, Deng C, Huang Y, Wang T (2021). Hollow-glass-microsphere-assisted half-circle interference for hydrostatic pressure measurement with high sensitivity. Opt. Express.

[79] Sun H, Liu X, Wang Z, Yu Y, Yang Y, Zhang X (2021). Temperature sensing characteristics of a microsphere resonator embedded in a capillary. J. Appl. Optics.

[80] Cai L, Pan J, Hu S (2020). Overview of the coupling methods used in whispering gallery mode resonator systems for sensing. Opt. Lasers Eng..

[81] Zhang S, Tang SJ, Feng S, Xiao YF, Cui W, Wang X, Sun W, Ye J, Han P, Zhang X, Zhang Y (2019). High-*Q* polymer microcavities integrated on a multicore fiber facet for vapor sensing. Adv. Opt. Mater..

[82] Huang H, Yu Y, Zhou L, Tao Y, Yang J, Zhang Z (2021). Whispering gallery modes in a microsphere attached to a side-polished fiber and their application for magnetic field sensing. Opt. Commun..

[83] Milenko K, Konidakis I, Pissadakis S (2016). Silver iodide phosphate glass microsphere resonator integrated on an optical fiber taper. Opt. Lett..

[84] Yan J, Wang D, Ge Y, Guo Y, Xu B (2022). A humidity sensor based on a whispering-gallery-mode resonator with an L-shaped open microcavity. J. Lightwave Technol..

[85] Niu P, Jiang J, Liu K, Wang S, Jing J, Xu T, Wang T, Liu Y, Liu T (2022). Fiber-integrated WGM optofluidic chip enhanced by microwave photonic analyzer for cardiac biomarker detection with ultra-high resolution. Biosens. Bioelectron..

[86] Deng B, Sima C, Tan H, Zhang X, Lian Z, Chen G, Yu Q, Xu J, Liu D (2021). Design of hollow core step-index antiresonant fiber with stepped refractive indices cladding. Front Optoelectron..

[87] Faruk M, Khan N, Biswas S (2020). Highly nonlinear bored core hexagonal photonic crystal fiber (BC-HPCF) with ultra-high negative dispersion for fiber optic transmission system. Front Optoelectron..

[88] Tsui N, Healy N (2021). Recent progress of semiconductor optoelectronic fibers. Front Optoelectron..

[89] Li J, Wang D (2022). A highly robust microsphere whispering-gallery-mode resonator in multimode fiber. Optik (Stuttg.).

[90] Hou F, Zhan Y, Feng S, Ye J, Wang X, Sun W, Zhang Y (2022). Smart grating coupled whispering-gallery-mode microcavity on tip of multicore optical fiber with response enhancement. Opt. Express.

[91] Li J, Wang D, Guo X (2022). Integrated in-fiber dual whispering gallery mode resonators device. IEEE Photonics Technol. Lett..

[92] Dai D (2009). Highly sensitive digital optical sensor based on cascaded high-*Q* ring-resonators. Opt. Express.

[93] Hodaei H, Miri MA, Heinrich M, Christodoulides DN, Khajavikhan M (2014). Parity-time–symmetric microring lasers. Science.

[94] Liu X, Xie Y, Chen Y, Wang Z, Yu J, Yang Y, Zhang X, Wang T (2021). Fiber coupled double microsphere resonator and its mode splitting characteristics. Acta Opt. Sin..

[95] Chen T, Zhang H, Lin W, Liu H, Liu B (2022). Highly sensitive refractive index sensor based on vernier effect in coupled micro-ring resonators. J. Lightwave Technol..

[96] Hulme JC, Doylend JK, Bowers JE (2013). Widely tunable Vernier ring laser on hybrid silicon. Opt. Express.

[97] Chen W, Kaya Özdemir Ş, Zhao G, Wiersig J, Yang L (2017). Exceptional points enhance sensing in an optical microcavity. Nature.

[98] Ma Z, Sun J, Zhou S, Shan W, Yan Y, Liu Y (2023). Compact fiber sensor for pH measurement based on the composite effect of hydrogel deformation and LC refractive index variation. Opt. Lett..

[99] He C, Sun H, Mo J, Yang C, Feng G, Zhou H, Zhou S (2018). Temperature sensor based on high-*Q* polymethylmethacrylate optical microbubble. Laser Phys..

[100] Wang X, Yu J, Zhao H, Lu X, Li W, Tian K, Brambilla G, Wang P (2020). 1.88 μm laser emission from Tm3+ doped fluorosilicate glass microspheres with excellent stability and high damage threshold. J. Lumin..

[101] Matsko A, Savchenkov A, Strekalov D, Ilchenko V, Maleki L (2005). Review of applications of whispering-gallery mode resonators in photonics and nonlinear optics. IPN Progress Report.

